# Inter-Varietal Diversity of Typical Volatile and Phenolic Profiles of Croatian Extra Virgin Olive Oils as Revealed by GC-IT-MS and UPLC-DAD Analysis

**DOI:** 10.3390/foods8110565

**Published:** 2019-11-09

**Authors:** Igor Lukić, Marina Lukić, Mirella Žanetić, Marin Krapac, Sara Godena, Karolina Brkić Bubola

**Affiliations:** 1 Institute of Agriculture and Tourism, Karla Huguesa 8, HR-52440 Poreč, Croatia; marina@iptpo.hr (M.L.); marin@iptpo.hr (M.K.); sara@iptpo.hr (S.G.); karolina@iptpo.hr (K.B.B.); 2 Centre of Excellence for Biodiversity and Molecular Plant Breeding, Svetošimunska 25, HR-10000 Zagreb, Croatia; 3 Institute for Adriatic Crops and Karst Reclamation, Put Duilova 11, HR-21000 Split, Croatia; mirella.zanetic@krs.hr

**Keywords:** extra virgin olive oil, volatile compounds, phenols, sensory quality, varietal typicity

## Abstract

Despite having an interesting native olive gene pool and a rapidly emerging olive oil industry, monovarietal extra virgin olive oils (EVOO) from Croatia are relatively unexplored. To investigate the inter-varietal diversity of typical volatile and phenolic profiles of Croatian EVOO, 93 samples from six olive (*Olea europaea* L.) varieties were subjected to gas chromatography-ion trap mass spectrometry (GC-IT-MS) and ultra-performance liquid chromatography with diode array detection (UPLC-DAD), respectively. Quantitative descriptive sensory analysis was also performed. Analysis of variance extracted many relevant exclusive or partial discriminators between monovarietal EVOOs among the identified volatile compounds and phenols. Successful differentiation model with a 100% correct classification was built by linear discriminant analysis, while the most typical volatiles for each monovarietal EVOO were confirmed by partial least squares discriminant analysis. Diverse typical sensory attributes among the EVOOs were tentatively ascribed to the variations in the composition of volatiles and phenols. It was proven that the approach that comprises GC-IT-MS and UPLC-DAD analysis may provide additional objective information about varietal origin and typicity which successfully complement those obtained by sensory analysis. The approach was characterized as universal in nature, with a significant potential to contribute in strengthening the varietal identities and position on the market of monovarietal and Protected Denomination of Origin (PDO) EVOO.

## 1. Introduction

Extra virgin olive oil (EVOO) is appreciated among consumers because of its specific flavor and nutritional properties. Due to its economic importance, EVOO is among the most common commodities subject to fraud and mislabeling. The European Union (EU) protects EVOO by the regulation mostly based on analytical and sensory controls [1] which generally succeed in detecting illegal manipulation with EVOO intrinsic properties (adulteration with cheaper refined and/or extraneous oils) and EVOO extrinsic properties (fraudulent misrepresentation of quality category). In the EU, most EVOOs of high economic value are additionally protected by Protected Denomination of Origin (PDO) [2]. Each PDO EVOO is produced according to a set of specific requirements prescribed by the holder of a designation in a specification document, governing aspects such as olive varieties used, cultivation, harvest and processing conditions, physico-chemical parameters, and sensory characteristics.

Many PDO EVOOs are produced from olives of a single variety (monovarietal EVOOs), while the blends also owe a large part of their typicity to the unique olive assortment of a particular region. Nowadays, the information on the label about the varietal origin of EVOO is becoming more and more important and attracting, especially for the market segment of informed consumers interested in healthy, quality products with remarkable diversity and clear identity. Similar as in the case of wine, besides being linked to a given geographical origin and PDO, EVOOs from particular varieties are recognized, appreciated, and demanded on the market because of their specific nutritional and sensory properties. As a consequence, they often reach higher prices, and, given the obvious financial benefits associated with them, are very likely subject to fraud by mislabeling with respect to varietal origin.

As regards EVOO varietal authentication within the process of protection by designation of origin (PDO), controls against counterfeiting include auditing of mandatory documentation and records that prove traceability in production and compliance with the requirements set up in PDO specification. The other part is the assessment of the conformity of EVOO with physico-chemical parameters and sensory characteristics laid down in the specification. The analytical parameters controlled more often (e.g., acidity, peroxide value, measurements in ultraviolet, etc.) do not specifically reflect varietal origin, and the limits that are established, although usually stricter than those prescribed by the official EU regulation [1], are regularly not designed to identify olive variety used. Similar applies for the sensory profiles commonly used to describe monovarietal/PDO EVOO [3] which are not highly discriminative.

The mentioned measures are not sufficient to control varietal origin and avoid fraud. Fraud or false labelling might also be detected or confirmed chemically by analysis of other, minor EVOO compounds. The general strategy that is followed in various research laboratories is the detection of as many as possible EVOO constituents from a larger set of samples and application of multivariate statistical analysis to the analytical data in order to build up classification/prediction models based on varietal origin [4,5]. Many EVOO compounds were found useful for this purpose, including sterols [6], tocopherols [7], fatty acids [6,8], etc. The chemical compounds whose amounts are regulated neither by the official EU regulation nor the PDO specifications, but are certainly the most involved in the typical sensory identity of PDO and monovarietal EVOOs and could serve as differentiators based on such criteria, are volatile aroma compounds and phenols [9,10,11,12,13,14]. In fact, many successful reports were published which confirmed the utility of these constituents for EVOO varietal differentiation [12,14,15,16,17,18,19,20]. Volatile fraction of high quality EVOO, which is responsible for its characteristic so-called *green* and *fruity* flavor, consists mainly of C5 and C6 volatiles (aldehydes, ketones, alcohols, and esters) generated enzymatically in the so-called lipoxygenase (LOX) pathway and other subsequent bioprocesses during olive processing. LOX-derived compounds are accompanied by those from other chemical classes, such as hydrocarbons, terpenes, benzenoids, etc. with mostly unknown or minor sensory relevance [12,14,21,22,23]. Besides being among the most important contributors to EVOO antioxidant activity, phenols, especially secoiridoids which are the most abundant, are responsible for the characteristic EVOO bitterness and pungency [12,13,14,24,25]. Olive oil phenols are formed mainly by cleavage of their glycosides by hydrolytic enzymes during olive fruit processing and their concentrations are further affected by oxidative degradation catalyzed by polyphenoloxidases and peroxidases [26,27]. The activity of the mentioned enzymes responsible for the formation of both volatile compounds and phenols is strongly genetically predetermined [28,29], which makes these compounds a logical choice for potential varietal markers in EVOO varietal characterization and differentiation studies.

Croatia is the latest country that joined EU in 2013, and some of the most recently registered PDOs are Croatian [30]. Despite relatively small quantities produced in relation to the leading olive oil producing countries, such as Spain, Italy, Greece etc. [31], EVOOs from Croatia are emerging rapidly on the global market and are much appreciated. For example, Croatian EVOOs are often among those awarded with the highest prizes at relevant international competitions, while Istria, one of the most important olive growing and EVOO producing regions in Croatia, has been represented in the first and leading global EVOO guide *Flos Olei* by the largest number of EVOOs among all the regions for the last four years in a row (2015–2018). The olive plantations in Croatia have high genetic diversity, including many native varieties which concentrate close to their area of origin and show a limited geographical dispersion [32]. For this reason, Croatian EVOOs protected by various PDOs owe a significant part of their typicity to the varietal origin of the olives, which certainly becomes most pronounced in the case of monovarietal EVOO. In spite of that, and despite existing reports on the chemical and sensory characteristics of Croatian monovarietal EVOO [33,34,35,36,37,38,39,40], the potential of Croatian native olive varieties to produce diverse and specific EVOO has not been investigated enough to be adequately exploited in designing more unique and robust PDOs.

The main aim of this study was to investigate the inter-varietal diversity of typical volatile and phenolic profiles of Croatian monovarietal EVOOs by gas chromatography-ion trap mass spectrometry (GC-IT-MS) and ultra-performance liquid chromatography with diode array detection (UPLC-DAD), respectively. The approach was tested for the characterization and differentiation of EVOOs made from native varieties grown in the two most important olive growing regions in Croatia, Istria and Dalmatia, with each monovarietal EVOO represented by a heterogeneous sample group in terms of geographical microlocations, growing conditions, harvest date, olive processing technology, and EVOO finalization and storage parameters. It was expected that the results obtained would be useful for improving the understanding of the origins of the typical sensory characteristics of the investigated Croatian monovarietal EVOOs. However, the main premise was that the instrumental techniques utilized would be effective in tracing robust chemical markers among the investigated compounds despite the aforementioned sample heterogeneity, able to provide complementary information about varietal origin to that obtained by sensory analysis. Besides allowing better quality management and control in production, such findings would contribute strengthening the PDO identities and position on the market of Croatian EVOO.

## 2. Materials and Methods

### 2.1. EVOO Samples

For this study, the most economically important and widespread Croatian native olive varieties (*Olea europaea* L.) were considered. Representative monovarietal EVOO samples, made from Buža (19 samples), Istarska bjelica (22 samples), and Rosinjola (8 samples) olive varieties specific for the region of Istria, and Oblica (15 samples) and Lastovka (10 samples) olive varieties specific for the region of Dalmatia, were collected from local producers. In addition, representative monovarietal EVOO samples from a widespread variety Leccino (19 samples) grown in Istria were also collected. Detailed climatological data for Istrian and Dalmatia regions in year 2015 are reported in Table S1. EVOOs were selected to cover the maximum possible variability of each production area, and all the samples from the same variety were produced by different producers. Olive fruit samples were hand-picked at the usual maturity level for each cultivar during the local customary harvest period during October/November 2015. The collected EVOO samples were produced in various private mills using contemporary oil extraction equipment with temperature of malaxation kept below 27 °C. After finalization (clarification and storage), market-ready EVOOs were kept at low temperature in amber dark glass bottles prior to analysis, and analyzed during a period of 3 months.

### 2.2. Sensory Analysis

Quantitative descriptive analysis of EVOO samples was performed by the Panel for sensory analysis of VOO of the Institute of Agriculture and Tourism in Poreč (Croatia), accredited for VOO sensory analysis according to the EN ISO/IEC 17025:2007 standard and authorized by the Croatian Ministry of Agriculture for official VOO testing from 2012, and recognized in continuation by the IOC from 2014. The panel consisted of eight assessors (4 female, 4 male, average age 39) trained and accredited for VOO sensory analysis according to the International Olive Council (IOC) method adopted by the European Commission Regulation [1]. As well, all the tasters have had long-term involvement in EVOO research and have gained large experience in Croatian monovarietal EVOO sensory analysis. Qualitative (selection of descriptors/attributes by consensus and standardization of vocabulary) and quantitative (intensity of perception) criteria of the tasters were attuned by audibly tasting representative samples of Croatian monovarietal EVOOs through several preliminary training sessions. The panel agreed that the sensory attributes which best describe the investigated monovarietal EVOOs were the same for all varieties and among those commonly perceived in EVOO, but differed with respect to the ratios of their intensities. The panel used a modified profile sheet expanded with particular positive odor and taste attributes, which were quantified using a 10 cm unstructured intensity ordinal rating scale from 0 (no perception) to 10 (the highest intensity). For evaluating general quality attributes, a 10-point overall structured rating scale from 0 (the lowest quality) to 10 (the highest quality) was applied. For overall quality evaluation, VOOs were graded with points from 1 (the lowest quality) to 9 (the highest quality). Before each session, the tasters attuned their criteria with respect to the intensities of the perceived sensory attributes by tasting the same standard reference VOO sample, a blend characterized by all the selected sensory attributes/descriptors. According to the sensory analysis, all the investigated samples were classified as EVOO (no defect, fruitiness > 0).

### 2.3. Chemical Standards and Standard Solutions

Methanol, water, and n-hexane were of HPLC grade purity (Sigma-Aldrich, St. Louis, MO, USA). Pure chemical standards of volatile compounds and phenols were purchased from AccuStandard Inc. (New Haven, CT, USA), Acros Organics (Geel, Belgium), Alfa Aesar (Haverhill, MA, USA), Cayman Chemical Co. (Ann Arbor, MI, USA), Extrasynthese (Genay, France), Fluka (Buchs, Switzerland), Honeywell International Inc. (Morris Plains, NJ, USA), Merck (Darmstadt, Germany), and Sigma-Aldrich. Standard solutions of volatiles were prepared in refined sunflower oil and that of phenols in pure methanol.

### 2.4. Analysis of Volatile Compounds by GC-IT-MS

Volatile compounds were isolated using headspace solid-phase microextraction (HS-SPME), according to the modified method proposed by Brkić Bubola, Koprivnjak, Sladonja, Škevin, and Belobrajić [41]. SPME fiber used was divinylbenzene/carboxen/polydimethylsiloxane (DVB/CAR/PDMS), 1 cm length, 50/30 μm film thickness (Supelco, Bellefonte, PA, USA). Four grams of EVOO sample (or a standard solution) were placed in a 10 mL glass vial containing a micro-stirring bar, and sealed. The headspace in the vial was equilibrated at 40 °C for 15 min, and the extraction was carried out at 40 °C for 40 min with stirring at 800 rpm. Thermal desorption of analytes was achieved in the GC injection port in splitless mode at 245 °C for 3 min. Identification and quantification of volatile compounds was performed using a Varian 3900 GC coupled to a Varian Saturn 2100 T ion trap mass spectrometer (IT-MS) (Varian Inc., Harbor City, CA, USA). A capillary column Rtx-WAX (60 m × 0.25 mm i.d. × 0.25 μm film thickness; Restek, Bellefonte, PA, USA) was used. Initial oven temperature was 40 °C, increased to 210 °C at 2 °C/min, increased to 245 °C at 20 °C/min, and kept for 20 min. Injector, transfer line and ion trap temperatures were 245, 180, and 120 °C, respectively. Mass spectra were acquired in EI mode (70 eV) at 1 s/scan, full scan with a range of 30–450 *m*/*z*. The carrier gas was helium (1.2 mL/min).

Identification was performed by comparing retention times and mass spectra with those of pure standards, and with mass spectra from NIST05 library. Identification by comparison with mass spectra was considered satisfactory if spectra reverse match numbers (RM) higher than 800 were obtained. If in a particular sample the mass spectra were not clear (RM < 800), identification was considered satisfactory if the ratios of a quantifier and three most abundant characteristic ions reasonably matched those in the reference spectra of a given compound. Linear retention indices (relative to C7–C24 n-alkanes) were calculated and compared to those from literature. When standards were available, standard calibration curves based on quantifier ions were used for quantification. Linearity was satisfactory with coefficient of determination higher than 0.99 for all the standards. For other compounds semi-quantitative analysis was carried out, and their concentrations (μg or mg/kg) were expressed as equivalents of the compounds with similar chemical structure for which standards were available, assuming a response factor equal to one.

### 2.5. Analysis of Phenols by UPLC-DAD

Extraction of phenols from EVOO was performed according to the modified method proposed by Jerman Klen, Golc Wondra, Vrhovšek, and Mozetič Vodopivec [42]. Ten grams of EVOO were dissolved in 10 mL of n-hexane, and 5 mL of methanol was added. The mixture was vortexed for 2 min, sonicated for 10 min, and then centrifuged at 5000 rpm for 5 min. The extraction was repeated 2 more times, and unified methanol extracts were defatted by 3 portions of 10 mL n-hexane. The methanol extracts (or standard solutions) were evaporated to dryness, the residue was re-dissolved in a 2 mL of a mixture of HPLC eluents (A (95:5 water—acetic acid (*v*/*v*)):B (methanol) = 90:10 (*v*/*v*)), and filtered through 0.45 μm PTFE filters.

Analysis of phenols was performed by ultra-performance liquid chromatography with diode array detection (UPLC-DAD) using an Agilent Infinity 1260 system (Agilent Technologies, Palo Alto, CA, USA) equipped with a G1311B quaternary pump, a G1329B autosampler, a G1316A column oven, and a G4212B DAD detector. A Kinetex PFP column (2.6 μm, 100 mm × 4.6 mm) with a guard was used (Phenomenex, Sydney, Australia) at 27 °C. Solvents were water with glacial acetic acid (95:5, *v*/*v*) (A) and methanol (B), with a flow rate of 1 mL/min. Ten microliters of the extract were injected. A 20-step gradient run used was reported previously [42]. Identification was performed by comparing retention times and UV/Vis spectra with those of pure standards when available, and with UV/Vis spectra from the literature [42]. Detection wavelengths were 280 nm (for simple phenols, vanillic acid, lignans, and secoiridoids), 320 nm (vanillin and *p*-coumaric acid), and 365 nm (flavonoids), while spectra were registered from 200 to 600 nm. Standard calibration curves were constructed for tyrosol, hydroxytyrosol, vanillic acid, vanillin, *p*-coumaric acid, luteolin, apigenin, pinoresinol, and oleuropein. For other compounds semi-quantitative analysis was carried out: secoiridoids were expressed in mg/kg as oleuropein, and acetoxypinoresinol as pinoresinol equivalents, respectively.

### 2.6. Statistical Data Elaboration

Data from GC-IT-MS, UPLC-DAD, and sensory analysis were subjected to one-way analysis of variance (ANOVA), and average values were compared by Least Significant Difference (LSD) test at the level of *p* < 0.05. Data were further processed by multivariate techniques, such as forward stepwise linear discriminant analysis (SLDA) and partial least squares discriminant analysis (PLSDA). The main goal of SLDA was to find the most useful variables (volatile compounds) for the mutual differentiation of all the six monovarietal EVOO. SLDA was applied on mean-centered data of a reduced dataset including six groups (varieties) and 50 variables with the highest *F*-ratios obtained in one-way ANOVA. Wilk’s lambda was used as a selection criterion with an *F* statistic factor to establish the significance of the changes in Lambda when a new variable is tested (*F*-value to enter = 1). The main goal of PLSDA was to find the most useful variables (volatile compounds) for the differentiation of each of the six investigated monovarietal EVOO from all the other (five) monovarietal EVOOs. For this reason, PLSDA was applied on mean-centered data of six separate datasets each including two groups (a single vs. other five monovarietal EVOOs) and all the 197 variables. Variable Importance in Projection (VIP) scores were determined as the weighted sums of the squares of the weight in the PLSDA. ANOVA and SLDA data elaboration were performed by Statistica v. 13.2 software (StatSoft Inc., Tulsa, OK, USA), while PLSDA analysis was conducted using MetaboAnalyst v. 4.0 (http://www.metaboanalyst.ca) created at the University of Alberta, Canada [43].

## 3. Results

### 3.1. Volatile Aroma Compound Profiles

A total of 197 volatile compounds were reported, including 29 hydrocarbons, 29 terpenes, 24 aldehydes, 11 ketones, 23 alcohols, 10 acids, 17 esters, 37 benzenoids, 8 furanoids, and 9 other compounds (Table 1). For many volatiles significant differences between average concentrations in the investigated EVOOs were found. Several volatile compounds emerged as exclusive markers of particular monovarietal EVOOs.

#### 3.1.1. Hydrocarbons

Istrian Buža and Rosinjola EVOOs stood out with the highest concentration of particular unsaturated hydrocarbons, such as several non-identified branched-chain alkenes, 3-ethyl-1,5-octadiene and 3,7-decadiene isomers, as well as that of saturated ones, such as decane, undecane, and dodecane (Table 1). On the other hand, lower amounts of the same groups of volatiles were found characteristic for Dalmatian Oblica and Lastovka, while I. bjelica EVOO contained intermediate concentrations. Similar relations were observed when comparing total hydrocarbons. Among hydrocarbons, 2,6-dimethyl-3-heptene turned out to be an exclusive marker of Rosinjola, and dodecene of Lastovka EVOO, respectively.

#### 3.1.2. Monoterpenes and Sesquiterpenes

Lastovka EVOO was distinguished by the highest concentrations of several monoterpenes, such as α-pinene, camphene, myrcene, β-phellandrene, and γ-terpinene, as well as total monoterpenes (Table 1). The same EVOO contained the highest levels of γ-elemene and particular non-identified sesquiterpenes. Several sesquiterpenes were characteristic for Oblica EVOO, with α-muurolene as the most prominent marker. The lowest concentrations of (+)-cycloisosativene, α-muurolene, δ-cadinene, and two unidentified sesquiterpenes, as well as total sesquiterpenes, were found in Lastovka and Leccino EVOOs.

#### 3.1.3. Aldehydes

Among unsaturated aldehydes formed in the so-called LOX pathway, Buža, followed by Rosinjola and Oblica EVOOs, contained the highest concentrations of (*E*)- and (*Z*)-3-hexenal, respectively (Table 1). The same monovarietal EVOOs, with sporadic exceptions, were also distinguished by high levels of pentenals, hexadienals, and (*E*)-2-octenal. Oblica EVOO had high concentration of decadienals. Leccino was clearly distinguished from the other monovarietal EVOOs by the highest level of the major EVOO volatile, (*E*)-2-hexenal, as well as that of (*E*,*E*)-2,4-heptadienal. Leccino EVOO had the lowest concentration of (*Z*)-3-hexenal, although not statistically different from that found in I. bjelica and Lastovka EVOOs. Lastovka EVOO was characterized by rather low levels of particular LOX-derived hexenals, as well as pentenals, octanal, and hexadienals. Istarska bjelica contained low concentrations of (*Z*)-3-hexenal and decadienals. Among saturated aldehydes originating from the processes other than LOX, 3-methylbutanal turned out to be an exclusive marker of I. bjelica, while abundance in 2-methyl-2-pentenal was observed in Rosinjola EVOO (Table 1). Higher levels of hexanal clearly discriminated Dalmatian (Oblica and Lastovka) from Istrian monovarietal EVOOs.

#### 3.1.4. Ketones

Istrian EVOOs contained higher concentration of the most important olive oil ketone in sensory terms, 1-penten-3-one, in relation to the Dalmatian ones, especially Lastovka (Table 1). The highest concentrations of 2-cyclohehene-1,4-dione were found in Rosinjola followed by Buža, while the lowest were found in Leccino EVOO. Dalmatian Oblica and Lastovka EVOOs were distinguished by the highest concentration of 1-(2,6,6-trimethyl-1-cyclohexene-1-yl)-1-penten-3-one and the lowest concentration of (*Z*)-cinerolone. The latter volatile compound was found to be a marker of Leccino, the same as 4′-ethoxy-2′-hydroxyoctadecanophenone was for Rosinjola EVOO.

#### 3.1.5. Alcohols

Among LOX-generated unsaturated C6 alcohols, a similar pattern as in the case of 3-hexenals was observed, with Leccino EVOO containing the lowest concentration of both (*E*)- and (*Z*)-3-hexen-1-ol (Table 1). Dalmatian EVOOs, especially Lastovka in the case of (*E*)-2-hexen-1-ol, exhibited the highest concentrations. 1-Penten-3-ol turned out to be a marker of I. bjelica EVOO, Rosinjola EVOO was the most abundant in 2-ethyl-1-hexanol and 2-(2-butoxyethoxy)-ethanol, while higher concentration of a number of non-LOX alcohols, such as 1-methoxy-2-propanol, 3-methyl-1-butanol, and 1-pentanol, turned out to be a feature of Lastovka EVOO.

#### 3.1.6. Acids

Leccino EVOO was characterized by the lowest concentration of (*E*)-3-hexenoic acid (Table 1). Dalmatian, especially Lastovka EVOO, had the highest concentrations of butanoic and hexanoic acids, while I. bjelica EVOO was by far the most abundant in other middle-chain volatile fatty acids, especially nonanoic acid, as well as total acids. Rosinjola was distinguished by higher levels of 2-ethylhexanoic acid, which corresponded well to the higher concentration of 2-ethyl-1-hexanol found in this EVOO.

#### 3.1.7. Esters

Istarska bjelica EVOO exhibited the highest concentration of the acetates of C6 alcohols, methyl acetate, and total esters (Table 1). Lastovka EVOO was abundant in hexyl acetate. Rosinjola EVOO stood out with the highest levels of several, mostly tentatively identified esters with high LRIs.

#### 3.1.8. Benzenoids

For many simple benzenoids (e.g., xylenes, ethylbenzenes, cymenes, etc.) no statistically significant differences between the monovarietal EVOOs were observed (Table 1). Rosinjola was the most distinguished by the highest concentrations of *p*-cymenene, acetophenone, 4-ethylbenzaldehyde, and the two non-identified aromatic aldehydes. Estragole and methyl anthranilate were found in the highest concentration in Lastovka EVOO, and these two compounds, together with lilial, 2-phenoxyethanol, and 4-ethoxystyrene, were more abundant in Dalmatian than in Istrian EVOOs. The highest concentration of benzyl nitrile was observed in Leccino EVOO. Low concentrations of several benzenoids were characteristic for particular EVOOs: methyl salicylate in I. bjelica, and methyl 2-methoxybenzoate and methyl anthranilate in I. bjelica and Leccino.

#### 3.1.9. Furanoids

Similar as for the benzenoids, Rosinjola EVOO was characterized by several furanoid markers, including 2-ethylfuran, 2-vinylfuran, and 5-methyl-2-furancarboxaldehyde (Table 1). On the other hand, Leccino EVOO had the lowest concentration of furanoids in general.

#### 3.1.10. Miscellaneous Compounds

Rosinjola EVOO contained the highest concentration of 2-phenyl-1H-indole and phenol (Table 1). Phenol concentration was the lowest in Dalmatian Oblica and Lastovka EVOOs.

#### 3.1.11. Odor Activity Values (OAV)

Table 2 lists the average odor activity values (OAV) of the volatile aroma compounds found in the investigated EVOOs, calculated as the ratios of their concentrations and odor perception thresholds available in literature. For thirteen compounds average OAV higher than 1 was observed in at least one of the monovarietal EVOOs implying their direct influence on the aroma. The compound with the highest OAV was (*Z*)-3-hexenal, followed by 1-penten-3-one and (*E*)-2-hexenal, while other compounds exhibited much lower OAVs. (*Z*)-3-hexenal was potentially the most important odorant in all the investigated monovarietal EVOOs except I. bjelica and Leccino in which 1-penten-3-one was dominant.

#### 3.1.12. Multivariate Statistical Analysis

**Figure 1:**
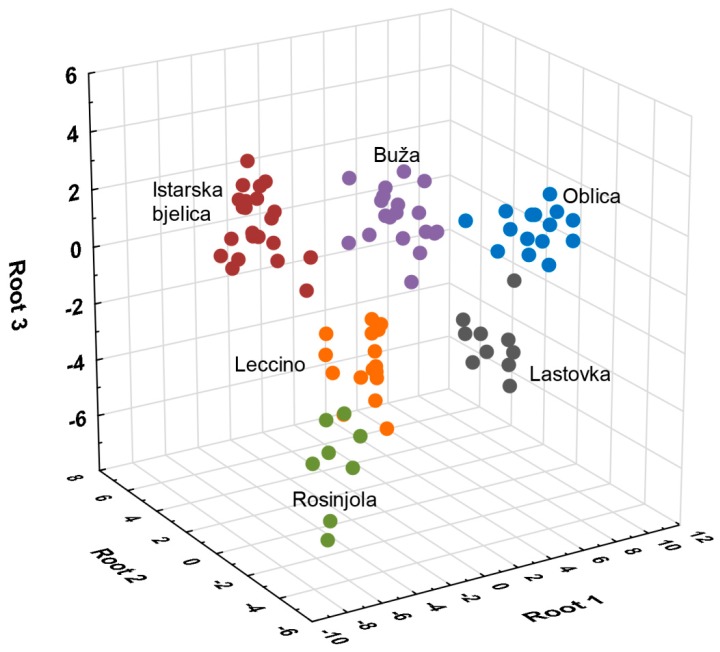
Separation of monovarietal extra virgin olive oils produced from Buža, Istarska bjelica, Rosinjola, Oblica, Lastovka, and Leccino varieties in Croatia according to variety in three-dimensional space defined by the first three discriminant functions (roots) on the basis of volatile aroma compound composition.

A differentiation model built by SLDA classified correctly all the monovarietal EVOOs according to variety (Figure 1) and extracted 30 variables (Table S2). A 100% correct classification was obtained after including 22 variables. Phenol was included in the model as the first and classified correctly 41.76% of all the investigated EVOO samples. After subsequently including nonanoic acid, α-muurolene, 3,7-decadiene I, and estragole (five compounds in total), the total percentage of the correctly classified EVOOs increased to 94.51%.

**Figure 2:**
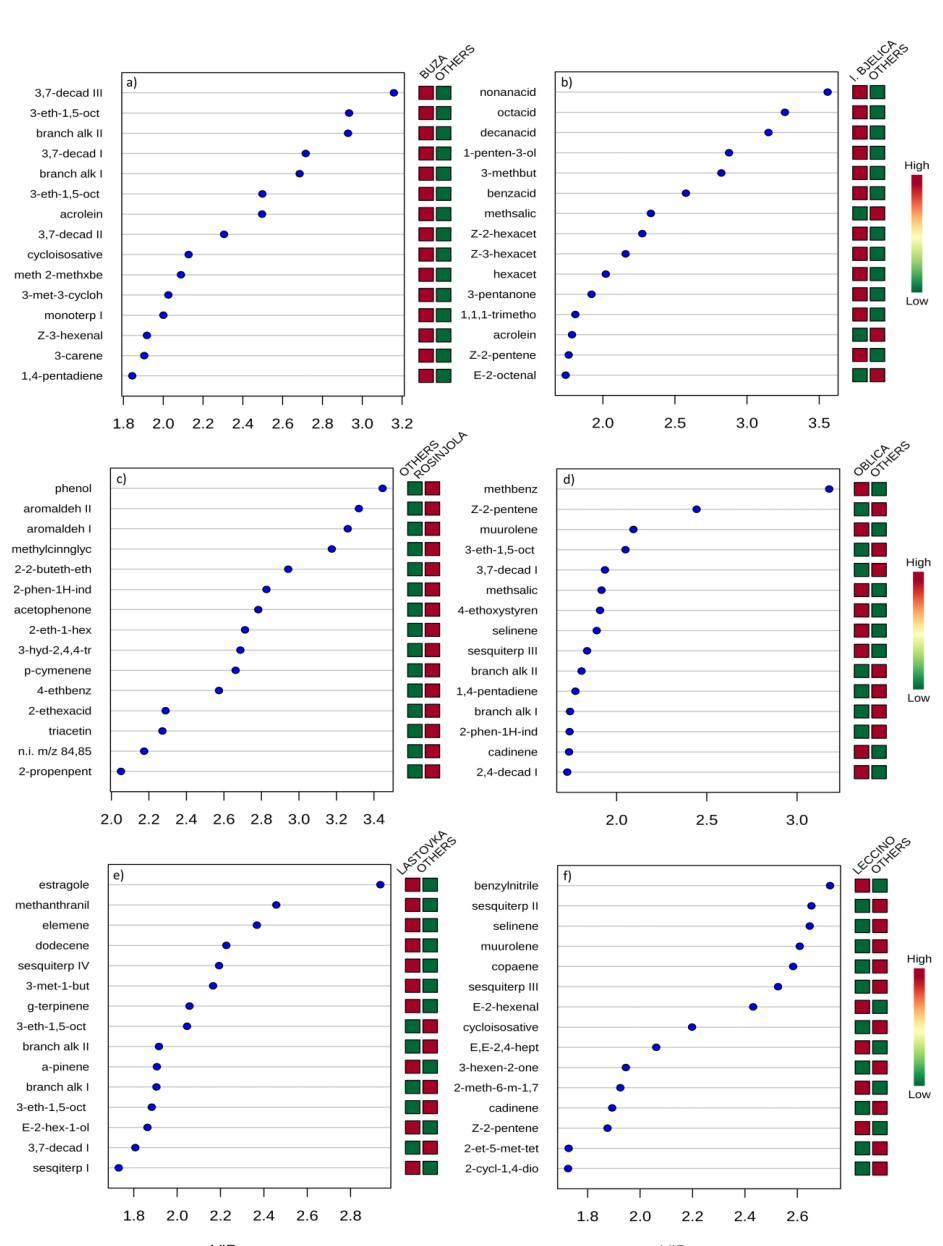
Variable Importance in Projection (VIP) scores of the variables (volatile compounds) most useful for the differentiation of each monovarietal extra virgin olive oil (EVOO), specifically: (**a**) Buža, (**b**) Istarska bjelica, (**c**) Rosinjola, (**d**) Oblica, (**e**) Lastovka, and (**f**) Leccino from the other five EVOOs produced in Croatia. Variables were extracted by partial least squares discriminant analysis applied on mean-centered data of six separate datasets each including two groups (a single vs. other five monovarietal EVOO).

Unsaturated hydrocarbons from the LOX pathway, accompanied by the most important LOX volatile (*Z*)-3-hexenal, were characterized by the highest positive VIP scores obtained by PLSDA and were confirmed to be typical for Buža EVOO (Figure 2a). Middle-chain fatty acids and hexenol acetates were the volatiles with the highest VIP scores in I. bjelica EVOO (Figure 2b), while the markers of Rosinjola EVOO were mostly benzenoids (Figure 2c). The volatile compound with by far the highest VIP score for the discrimination of Oblica was methyl benzene (Figure 2d), while Lastovka EVOO typicity was mostly owed to its abundance in terpenes and deficiency in LOX volatiles (Figure 2e). High concentrations of benzyl nitrile, (*E*)-2-hexenal, and (*E*,*E*)-2,4-heptadienal, as well as low concentrations of sesquiterpenes, were confirmed to be the most prominent typical characteristics of the Leccino EVOO volatile profile (Figure 2f).

### 3.2. Phenols

Nineteen phenolic compounds were identified in total, including simple phenols, phenolic acids, flavonoids, lignans, and secoiridoids (Table 3). For many of those significant differences between average concentrations in the investigated EVOOs were found, and a few phenols emerged as exclusive markers of particular monovarietal EVOOs. Lastovka EVOO was generally characterized by the highest concentrations of simple phenols (except vanillin) and *p*-coumaric acid. Leccino turned out to be clearly distinguishable from the other monovarietal EVOOs by the highest concentration of vanillin, and the lowest concentrations of *p*-coumaric acid and luteolin. Among lignans, high pinoresinol content was characteristic for Buža EVOO. The secoiridoid profiles differed among the investigated monovarietal EVOOs. The concentration of one of the major oleuropein aglycons and phenols in general in olive oil, dialdehydic form of decarboxymethylelenolic acid linked to hydroxytyrosol, i.e., 3,4-DHPEA-EDA or oleacein, was the highest in Leccino EVOO. Its tyrosol-based analogue, the major aglycon of ligstroside, *p*-HPEA-EDA or oleocanthal, clearly distinguished two groups of EVOO, I. bjelica, Oblica, and Leccino with higher, and Buža, Rosinjola, and Lastovka EVOOs with lower concentrations. The composition of other oleuropein and ligstroside aglycons also turned out to be variety-specific; it is worth mentioning low concentration of oleuropein aglycon I in Oblica and Leccino, low concentration of oleuropein aglycon II in Oblica, and exceptionally higher concentration of oleuropein + ligstroside aglycons in I. bjelica than in other EVOOs.

### 3.3. Sensory Attributes

**Figure 3:**
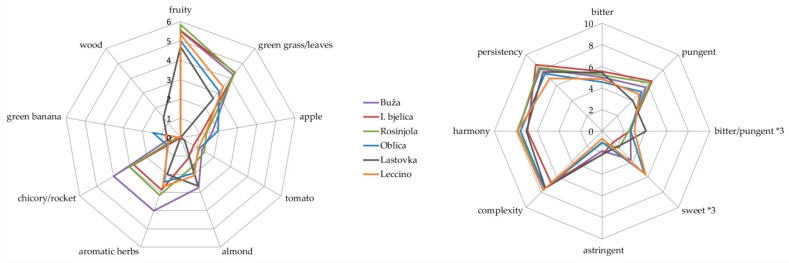
The intensities of the sensory attributes obtained by quantitative descriptive sensory analysis of monovarietal extra virgin olive oils (EVOOs) produced from Buža, Istarska bjelica, Rosinjola, Oblica, Lastovka, and Leccino varieties in Croatia (* 3 – the intensities of particular sensory attributes were multiplied by 3 to better visualize the differences between monovarietal EVOOs).

The majority of the investigated monovarietal EVOOs were characterized by common EVOO sensory attributes (Figure 3, Table S3). Buža EVOO showed higher intensities of the majority of the assessed positive odor attributes, and it was clearly distinguished from the others by the highest intensity of chicory/rocket. Istarska bjelica and Rosinjola had the lowest intensity of almond. The specificity of the odor of Oblica EVOO was contained mainly in the most intense green banana nuance, while Lastovka was distinguished as the only monovarietal EVOO with the woody note. Istrian EVOOs were generally described by higher intensities of green grass/leaves, aromatic herbs, and chicory/rocket attributes (with the exception of Leccino EVOO) in relation to the Dalmatian ones.

As regards the main EVOO taste attributes, bitterness and pungency, the EVOOs from Istrian native varieties Buža, I. bjelica, and Rosinjola generally showed higher intensities. The exception was the intensity of bitterness in Lastovka which was among the highest, which resulted in the highest bitterness/pungency ratio in this EVOO (Figure 3, Table S3). Oblica and Leccino were described as the sweetest among the EVOOs.

Istarska bjelica EVOO was characterized as the least complex, while I. bjelica and Lastovka EVOOs were less harmonious with respect to others. EVOOs from Istrian native varieties were the most, and Leccino the least persistent in terms of pungency.

## 4. Discussion

From the results of the GC-IT-MS and UPLC-DAD analysis of volatile compounds and phenols, respectively, it was clear that each of the investigated monovarietal EVOOs was characterized by a unique volatile and phenol profile. Since the samples were collected from various producers and were relatively heterogeneous in terms of geographical microlocations, growing conditions, harvest date, olive processing technology, and EVOO finalization and storage parameters, it could be assumed, with a high degree of certainty, that the effects of all of these factors were random, and that varietal origin was the main source of the observed differences. In fact, varietal origin was previously found to have a greater impact on volatile composition than various environmental factors [28]. However, the geographical origin possibly had an effect, which was impossible to evaluate separately from the effect of variety considering the varieties studied were specific for their regions. Istrian EVOOs, mostly those made of native Buža and Rosinjola, as well as those of the international variety Leccino, were characterized by higher concentrations of many LOX volatiles in relation to Dalmatian Oblica and Lastovka EVOO, including the most odoriferous ones, such as hexenals and 1-penten-3-one (Table 1). It is probable that this was directly reflected on the differences in their sensory profiles, since Istrian EVOOs had higher intensities of the majority of positive odor attributes, especially those of *green grass*/*leaves* and *chicory*/*rocket* (Figure 3, Table S3). Lower OAV values of the most potent odorants, (*Z*)-3-hexenal, 1-penten-3-one, and (*E*)-2-hexenal found in the Dalmatian, especially Lastovka EVOO, corroborated this assumption (Table 2). Since Istria is a region characterized by lower average temperatures than Dalmatia (Table S1), these results basically corroborated what was previously found in the majority of such studies that the temperature of environment is negatively correlated with the concentrations of LOX-derived volatile aroma compounds and the resulting EVOO positive sensory attributes [48,49].

Although without statistical significance in some cases, Buža EVOO excelled with the highest concentrations of the majority of positive LOX volatiles (Table 1), as well as with the highest intensities of positive odor sensory attributes (Figure 3, Table S3), which were probably in a causal relationship. It is worth emphasizing the highest cumulative odor activity value (OAV) of (*Z*)-3-hexenal and 1-penten-3-one, the two most powerful known odorants in EVOO with very low odor perception thresholds of 0.0017 and 0.00073 mg/kg, respectively [22,46] (Table 2), which certainly exhibited key roles. A large proportion of LOX volatiles among those extracted by the PLSDA as the most significant VIP compounds discriminating Buža from the other EVOOs (Figure 2) corroborated the assumption that this variety is characterized by strong lipoxygenase and hydroperoxide lyase activities in the LOX pathway. 

Generally, the most similar to Buža in terms of high concentrations of LOX volatiles (Table 1) and their OAVs (Table 2), as well as high intensities of positive odor attributes (Figure 3, Table S3), was Rosinjola EVOO. When it came down to the discriminating VIP compounds, those extracted by PLSDA were mostly benzenoids (Figure 2). Many benzenoids which were found in relatively high concentration in Rosinjola EVOO, including methyl benzoate, acetophenone, and methyl salicylate, were previously reported to be important almond odorants [50], however their impact in olive oil has not been investigated yet. As well, *almond* note was not especially accentuated in Rosinjola EVOO (Figure 3, Table S3).

Istarska bjelica had lower concentrations of many important LOX volatiles (Table 1). Since it is a late ripening variety [51] it is possible that it was characterized by a slightly weaker LOX enzymatic load with respect to Buža and Rosinjola EVOO. As it is known that phenols may act as LOX enzymatic activity inhibitors [52], the possibility that the high concentrations of phenols found in this monovarietal EVOO (Table 3) acted in this way during milling and malaxation should not be excluded. However, the concentrations and OAVs of some other major LOX odorants, such as (*E*)-2-hexenal and 1-penten-3-one, were relatively high, suggesting a notable activity of (*Z*)-3:(*E*)-2-enal isomerase which catalyzes the conversion of (*Z*)-3- to (*E*)-2-hexenal, as well as relatively high activity of the enzymes or availability of the substrates involved in the synthesis of C5 compounds via 13-alkoxy radicals in this side-branch of the LOX pathway. The concentrations and OAVs of these volatiles were not lower that those found in Buža and Rosinjola EVOOs (Table 1 and Table 2), so it is probable that (*E*)-2-hexenal and 1-penten-3-one were the key odorants in the formation of I. bjelica aroma and were the most responsible for the high intensity of several positive sensory attributes observed in this EVOO (Figure 3, Table S3). The most typical VIP chemical markers distinguishing I. bjelica EVOO were mostly non-LOX volatiles, namely middle-chain fatty acids and C6 alcohol acetates (Figure 2). Judging on the determined OAV values (Table 2), their sensory relevance was probably minor to medium. High concentrations of C6 alcohol acetates (Table 1) implied a possible high alcohol acyl transferase activity in olives and olive paste of this variety [53].

As stated previously, Oblica was characterized by a slightly lower contribution of the LOX volatiles and, consequently, lower intensities of particular positive odor attributes with respect to Istrian EVOOs, but was still superior to Lastovka EVOO (Table 1, Figure 3, Table S3). It was possibly mostly due to lower 1-penten-3-one and (*E*)-2-hexenal concentrations and OAVs, since the level of (*Z*)-3-hexenal was relatively high (Table 1 and Table 2). As well, it is possible that a part of the *fruity* and *green* aroma originated from hexanal, found in higher concentration with respect to the Istrian EVOOs (Table 1 and Table 2). *Green banana* odor sensory attribute which was found to be typical for Oblica EVOO (Figure 3, Table S3) could have, at least partly, originated from the volatiles often associated with this nuance. (*Z*)-3-hexen-1-ol was certainly a candidate for this role [45], since its concentration was the highest in this EVOO (Table 1) and at the same time above the corresponding odor detection threshold (Table 2). For other LOX volatiles with the odor commonly described as banana-like, such as hexanol, hexenyl acetates and penten-1-ols [45,54], no significant differences between varieties were found. As well, their levels in Oblica were not among the highest among the investigated EVOOs (Table 1), implying their impact in the formation of *green banana* nuance was probably not crucial. The same applies for other minor volatiles commonly reported as carriers of banana odor, such as isoamyl and other acetates. The VIP compounds responsible for the differentiation of Oblica EVOO (Figure 2), which pertained to several chemical families, could have not been meaningfully related to the occurrence of *green banana* odor.

Lastovka EVOO was characterized by the most distinguishable volatile profile among the investigated monovarietal EVOOs. It contained the lowest concentrations of the majority of LOX volatiles (Table 1), including the most potent odorants with the highest OAVs (Table 2), which was certainly a direct cause of the lowest intensities of the majority of positive odor attributes perceived in this EVOO (Figure 3, Table S3). On the other hand, it was found to have high amounts of hexanal, particular C6 alcohols, and hexyl acetate, compounds often accounted among the carriers of *green* odor which derive from the enzymatic degradation of linoleic acid but also oxidation [10,28,55]. Lastovka EVOO contained the highest concentrations of particular monoterpenes and sesquiterpenes, whose sensory contribution is generally described by descriptors such as *citrus*, *camphor*, *eucalyptus*, *roses*, etc., as well as *wood*. Although sensory relevance of terpenes in olive oil is currently still unknown and it is certainly limited by the lipid matrix in which these lipophilic molecules are highly soluble, the possibility of their contribution to the specific *wood* odor perceived in Lastovka EVOO during sensory analysis (Figure 3, Table S3) should not be excluded. Several terpenes were extracted by PLSDA as among the most discriminative compounds for this variety (Figure 2). Particular sesquiterpenes, on the other hand, such as (+)-cycloisosativene, α-copaene, α-muurolene, δ-cadinene, and several unidentified ones were found in the lowest concentrations in Lastovka EVOO (Table 1). Terpenes were previously found to have large potential to differentiate EVOO according to variety [16,56], which was basically confirmed in this study. Other compounds found to be characteristic for Lastovka EVOO, such as particular saturated short-chain aldehydes, ketones, alcohols, and acids, could also have had a sensory impact with their *malty*, *pungent*, *rancid*, and *sweaty* nuances (Table 2).

One of probably the most important characteristics found typical for Leccino EVOO was the ratio between the important (*E*)-2- and (*Z*)-3-C6 forms, which was generally the highest and discriminated well this EVOO from the majority of the other studied EVOOs (Table 1 and Table 2). The highest concentration of (*E*)-2-hexenal and the lowest concentration of (*Z*)-3-hexenal, (*Z*)-3-hexen-1-ol, as well as the low concentration of (*Z*)-3-hexenyl acetate in Leccino EVOO were likely the result of high (*Z*)-3:(*E*)-2-enal isomerase activity in Leccino olives, i.e., olive paste during milling and malaxation steps [57]. Considering that the estimated contribution of (*E*)-2-hexenal to the aroma of EVOOs was generally lower than that of (*Z*)-3-hexenal (Table 2), it is possible that one of the consequences of the observed differences was a slightly lower intensity of particular odor sensory attributes, such as *green*/*grass leaves* and *chicory*/*rocket*, observed in Leccino with respect to the EVOOs from the other, native Istrian varieties Buža, I. bjelica and Rosinjola (Figure 3, Table S3). Other interesting features of Leccino EVOO included lower concentrations of particular sesquiterpenes and furanoids. In fact, many sesquiterpenes were among those with the highest VIP scores extracted by PLSDA, but with a negative sign (Figure 2).

Phenols, especially secoiridoids, are responsible for the characteristic EVOO bitterness and pungency, but the specific sensory contribution of each individual major secoiridoid has not been precisely elucidated up to date. Nevertheless, there is solid evidence that *p*-HPEA-EDA is a key contributor to pungency, while the pungency of other, monoaldehydic ligstroside aglycons is weaker, although still strong [24]. Ligstroside aglycons were found to generally be less bitter than pungent, which was especially the case for *p*-HPEA-EDA. In the same study [24] it was found that the majority of oleuropein aglycons, including 3,4-DHPEA-EDA, was described as both bitter and pungent, with some of them exhibiting rather strong bitterness. The lowest intensity of bitterness observed in Oblica and Leccino (Figure 2, Table S3) could be tentatively linked to the lowest oleuropein aglycon I concentrations found in these EVOO (Table 3). The intensity of pungency did not quite correlate with the average *p*-HPEA-EDA concentrations (Figure 3, Table 3, Table S3). In contrast to I. bjelica, for which a positive correlation was observed, Rosinjola EVOO was characterized as intensively pungent according to the official method [1] (intensity >6) despite containing relatively low concentration of this secoiridoid. The highest average concentration of oleuropein aglycon II in Rosinjola EVOO (Table 3) could have possibly compensated for this deficiency. The pungency of I. bjelica possibly partly originated also from the highest concentrations of all the three monoaldehydic ligstroside aglycons found in this EVOO (Table 3). Especially interesting was the highest ratio of bitterness to pungency found in Lastovka EVOO (Figure 3, Table S3). Roughly, Lastovka EVOO contained among the highest concentrations of oleuropein aglycones and among the lowest concentrations of ligstroside aglycones, which could have had such an impact. This EVOO had the highest concentration of *p*-coumaric acid which, although relatively low, possibly contributed to the bitterness observed. It is worth mentioning that the UPLC chromatograms of Lastovka EVOO contained several unidentified peaks in addition to those observed in the other monovarietal EVOOs (data not shown), which possibly originated from the compounds with sensory relevance. The so-called *sweetness* in most cases coincided with the lower amounts of total phenols, which was as expected (Figure 3, Table 3). Again, several features turned out to be specific for Leccino EVOO (Table 3), the most important being the highest concentration of 3,4-DHPEA-EDA, which implied variety-dependent differences with respect to the availability of precursors and enzymatic activity between Leccino and Croatian native olive varieties.

## 5. Conclusions

The use of GC-IT-MS and UPLC-DAD proved to be a powerful combination for studying the inter-varietal diversity of typical volatile and phenolic profiles of Croatian EVOOs, respectively. Each of the investigated monovarietal EVOO displayed unique volatile aroma and phenol composition. The qualitative and quantitative chromatographic data was useful for tentative elucidation of some of the perceived sensory attributes including the variety-typical ones, which though has to be taken with caution due to the extreme complexity of the established chemical profiles with the majority of volatiles with still unknown sensory relevance. Many potential varietal markers were extracted by uni- and multivariate statistical analysis despite high intra-varietal heterogeneity. It was demonstrated that volatiles and phenols from all the investigated chemical classes can be useful for this purpose. Many of the volatile compounds which turned out to have a notable discrimination power were (tentatively) identified for the first time in EVOO, or were generally neglected in previous studies, especially from sensorial point of view. In fact, only in a few cases were the major LOX compounds, studied most extensively among the volatiles up to date, sufficient for a robust varietal differentiation in this work. This indicates a large potential of the untargeted fingerprinting approach for EVOO characterization, differentiation, and authentication studies.

The number of the extracted robust varietal markers among the investigated chemical compounds largely exceeded the number of typical sensory attributes useful to differentiate monovarietal EVOOs. It is reasonable to conclude that the approach which comprises GC-IT-MS and UPLC-DAD analytical techniques may provide additional objective information about varietal origin which successfully complement those obtained by sensory analysis. Probably the best example for this is the case of Rosinjola EVOO which was relatively similar and hardly distinguishable from that of Buža variety based solely on the sensory analysis, but was characterized by many exclusive chemical markers among benzenoid and furanoid volatiles which discriminated this EVOO rather successfully. 

The results obtained in this study could certainly be useful for improving the quality management and control in the production of Croatian monovarietal/PDO EVOO. These findings could contribute to strengthening their PDO identities and position on the market, and could be especially useful for discriminating EVOOs of Croatian native varieties from the world famous Leccino variety.

## Figures and Tables

**Table 1:** Concentrations (μg/kg unless otherwise stated) of volatile aroma compounds determined by gas chromatography-ion trap mass spectrometry (GC-IT-MS) after headspace solid-phase microextraction (HS-SPME) from monovarietal extra virgin olive oils produced from Buža, Istarska bjelica, Rosinjola, Oblica, Lastovka, and Leccino varieties in Croatia.

Compound	ID	LRI	Variety					
			Buža	I. bjelica	Rosinjola	Oblica	Lastovka	Leccino
*hydrocarbons*								
propane	MS	<700	6.64 ^bc^	14.91 ^a^	1.12 ^c^	11.49 ^ab^	12.51 ^a^	5.43 ^c^
(*Z*)-2-pentene	MS	<700	61.19 ^b^	80.27 ^a^	60.31 ^b^	33.98 ^c^	43.63 ^c^	83.09 ^a^
1,4-pentadiene (mg/kg)	LRI,MS	<700	0.17 ^a^	0.16 ^a^	0.15 ^ab^	0.10 ^c^	0.09 ^c^	0.12 ^b^
1,3-pentadiene	LRI,MS	<700	81.73 ^a^	75.36 ^ab^	71.98 ^ab^	53.80 ^c^	48.98 ^c^	62.92 ^bc^
1-heptene	LRI,MS	731	5.42	16.64	3.25	9.76	5.72	6.13
3,5-dimethylheptane	MS	753	1.61	3.59	3.63	2.38	0.33	2.86
propanal	LRI,MS	778	7.00	5.23	7.36	5.10	5.68	7.04
octane	S,LRI,MS	800	31.03	35.33	27.29	30.78	40.92	27.11
2,6-dimethyl-3-heptene	MS	821	0.20 ^b^	0.58 ^b^	6.17 ^a^	0.00 ^b^	0.00 ^b^	0.19 ^b^
(*E*)-2-octene	LRI,MS	832	3.50	4.60	3.81	3.36	5.62	3.60
branched-chain alkene I (n.i.)	MS	953	94.32 ^a^	63.73 ^c^	85.98 ^ab^	47.29 ^d^	38.96 ^d^	74.97 ^bc^
branched-chain alkene II (n.i.)	MS	960	84.13 ^a^	56.70 ^c^	71.63 ^b^	41.67 ^d^	35.22 ^d^	65.24 ^bc^
decane	S,LRI,MS	997	31.53 ^ab^	19.98 ^bc^	39.92 ^a^	9.49 ^c^	7.39 ^c^	14.67 ^c^
3-ethyl-1,5-octadiene I (mg/kg)	LRI,MS	1006	0.36 ^a^	0.26 ^c^	0.34 ^ab^	0.18 ^d^	0.15 ^d^	0.31 ^b^
3-ethyl-1,5-octadiene II (mg/kg)	LRI,MS	1020	0.40 ^a^	0.25 ^c^	0.35 ^ab^	0.20 ^d^	0.16 ^d^	0.31 ^b^
3-ethyl-1,5-octadiene III	LRI,MS	1033	2.99	12.91	3.19	1.24	0.79	2.60
branched-chain alkene III (n.i.)	MS	1067	0.37	10.08	0.45	0.06	0.01	0.48
3,7-decadiene I	LRI,MS	1074	76.81 ^a^	44.98 ^c^	71.69 ^ab^	33.06 ^d^	29.07 ^d^	65.14 ^b^
branched-chain alkene IV (n.i.)	MS	1077	3.59	6.51	1.87	1.62	0.72	2.92
3,7-decadiene II (mg/kg)	LRI,MS	1080	0.29 ^a^	0.17 ^b^	0.28 ^a^	0.15 ^bc^	0.13 ^c^	0.26 ^a^
3,7-decadiene III (mg/kg)	LRI,MS	1084	0.29 ^a^	0.15 ^c^	0.25 ^ab^	0.15 ^c^	0.11 ^c^	0.21 ^b^
branched-chain alkene V (n.i.)	MS	1087	7.98 ^ab^	11.87 ^a^	6.30 ^ab^	4.05 ^b^	3.00 ^b^	5.80 ^b^
undecane	S,LRI,MS	1095	18.56 ^ab^	9.75 ^b^	39.12 ^a^	3.29 ^b^	2.09 ^b^	9.83 ^b^
dodecane	S,LRI,MS	1203	23.4 ^ab^	14.16 ^bc^	35.51 ^a^	4.64 ^c^	2.64 ^c^	13.66 ^bc^
dodecene	LRI,MS	1241	27.26 ^bc^	33.95 ^b^	17.25 ^cd^	14.74 ^cd^	58.52 ^a^	8.43 ^d^
3-propylcyclohexene	MS	1247	10.97 ^bc^	7.93 ^c^	14.15 ^ab^	13.41 ^ab^	9.90 ^bc^	14.85 ^a^
1,5,5,6-tetramethyl-1,3-cyclohexadiene	LRI,MS	1365	2.46 ^bc^	1.55 ^c^	4.38 ^abc^	5.73 ^a^	5.52 ^ab^	1.82 ^c^
2,2-dimethyl-(*Z*)-3-hexene I	MS	1499	28.41 ^a^	16.09 ^b^	33.60 ^a^	26.67 ^a^	14.60 ^b^	10.76 ^b^
2,2-dimethyl-(*Z*)-3-hexene II	MS	1560	4.79	2.12	6.05	4.78	2.39	2.88
*monoterpenes*								
α-pinene	S,LRI,MS	1015	6.28 ^b^	4.91 ^b^	12.25 ^b^	8.90 ^b^	41.71 ^a^	2.79 ^b^
camphene	S,LRI,MS	1053	0.23 ^b^	0.10 ^b^	0.25 ^b^	0.31 ^b^	1.35 ^a^	0.04 ^b^
β-pinene	S,LRI,MS	1099	0.41	0.77	1.98	1.10	83.74	0.46
sabinene	S,LRI,MS	1112	2.07	0.79	2.09	2.94	49.44	17.20
3-carene	S,LRI,MS	1139	4.72 ^a^	3.06 ^b^	4.20 ^ab^	0.97 ^c^	2.29 ^bc^	0.83 ^c^
monoterpene I (n.i.)	MS	1140	2.77 ^a^	1.84 ^b^	2.54 ^ab^	0.40 ^c^	1.35 ^bc^	0.27 ^c^
myrcene	S,LRI,MS	1157	8.60 ^b^	4.36 ^b^	19.76 ^ab^	11.48 ^b^	41.67 ^a^	11.85 ^b^
α-terpinene	S,LRI,MS	1171	0.37	0.23	1.12	0.72	10.84	9.91
limonene (mg/kg)	S,LRI,MS	1191	0.02	0.08	0.02	0.02	0.64	0.01
β-phellandrene	LRI,MS	1201	0.00 ^b^	0.00 ^b^	0.00 ^b^	0.03 ^b^	4.25 ^a^	0.02 ^b^
(*Z*)-ocimene	S,LRI,MS	1230	15.14 ^b^	8.00 ^b^	25.27 ^ab^	34.84 ^a^	37.26 ^a^	10.72 ^b^
γ-terpinene	S,LRI,MS	1238	30.50 ^b^	4.96 ^b^	4.17 ^b^	3.74 ^b^	436.30 ^a^	40.80 ^b^
(*E*)-ocimene (mg/kg)	S,LRI,MS	1245	0.21 ^bc^	0.09 ^c^	0.27 ^abc^	0.52 ^a^	0.43 ^ab^	0.12 ^c^
monoterpene II (n.i.)	MS	1260	0.69 ^bc^	0.40 ^c^	1.28 ^abc^	1.82 ^a^	1.49 ^ab^	0.53 ^bc^
terpinolene	S,LRI,MS	1275	0.44	0.28	0.70	0.38	3.83	0.84
(*Z*)-alloocimene	LRI,MS	1349	11.38	9.68	10.20	23.02	8.22	7.20
(*E*)-alloocimene	S,LRI,MS	1357	16.79	12.63	13.25	37.22	10.66	9.26
linalool	S,LRI,MS	1536	15.44 ^bc^	8.94 ^c^	19.00 ^abc^	31.43 ^ab^	40.98 ^a^	22.04 ^abc^
*sesquiterpenes*								
(+)-cycloisosativene (mg/kg)	LRI,MS	1477	1.15 ^a^	0.87 ^b^	0.80 ^b^	0.89 ^ab^	0.24 ^c^	0.26 ^c^
α-copaene (mg/kg)	LRI,MS	1487	8.44 ^a^	7.02 ^ab^	5.81 ^b^	8.77 ^a^	2.14 ^c^	1.64 ^c^
sesquiterpene I (n.i.)	MS	1536	0.00 ^b^	0.00 ^b^	0.00 ^b^	0.00 ^b^	3.44 ^a^	0.00 ^b^
sesquiterpene II (n.i.) (mg/kg)	MS	1583	0.29 ^a^	0.27 ^a^	0.23 ^a^	0.28 ^a^	0.08 ^b^	0.05 ^b^
sesquiterpene III (n.i.) (mg/kg)	MS	1683	0.11 ^ab^	0.09 ^b^	0.09 ^b^	0.13 ^a^	0.03 ^c^	0.02 ^c^
δ-selinene	LRI,MS	1698	24.52 ^b^	25.84 ^ab^	10.32 ^c^	32.29 ^a^	6.82 ^c^	2.07 ^c^
γ-elemene (mg/kg)	MS	1704	0.04 ^b^	0.05 ^b^	0.05 ^b^	0.06 ^b^	0.20 ^a^	0.05 ^b^
α-muurolene (mg/kg)	LRI,MS	1719	1.65 ^b^	1.42 ^b^	1.26 ^b^	2.10 ^a^	0.49 ^c^	0.34 ^c^
sesquiterpene IV (n.i.)	MS	1736	0.63 ^c^	3.02 ^c^	26.21 ^b^	2.33 ^c^	51.54 ^a^	29.75 ^b^
α-farnesene (mg/kg)	LRI,MS	1745	0.53 ^a^	0.29 ^b^	0.84 ^a^	0.12 ^b^	0.25 ^b^	0.17 ^b^
δ-cadinene (mg/kg)	LRI,MS	1750	0.14 ^ab^	0.11 ^b^	0.17 ^a^	0.17 ^a^	0.06 ^c^	0.07 ^c^
*aldehydes*								
acrolein	LRI,MS	829	6.07 ^a^	2.34 ^d^	4.66 ^ab^	4.23 ^bc^	2.60 ^cd^	3.75 ^bc^
2-methylbutanal	LRI,MS	903	10.74	13.39	11.93	7.86	18.70	13.76
3-methylbutanal	S,LRI,MS	906	10.51 ^b^	22.29 ^a^	10.65 ^b^	8.49 ^b^	12.31 ^b^	12.26 ^b^
2-methyl-2-pentenal	MS	930	1.08 ^c^	0.26 ^cd^	3.34 ^a^	2.15 ^b^	0.85 ^cd^	0.00 ^d^
(*E*)-2-butenal	S,LRI,MS	1024	11.44	9.98	12.87	9.10	18.39	25.45
unsaturated aliphatic aldehyde I (n.i.)	MS	1036	3.27 ^a^	2.25 ^b^	2.63 ^ab^	2.48 ^ab^	1.56 ^b^	1.68 ^b^
unsaturated aliphatic aldehyde II (n.i.)	MS	1051	0.25	5.31	0.55	0.03	0.04	0.25
hexanal (mg/kg)	S,LRI,MS	1070	0.24 ^b^	0.29 ^b^	0.27 ^b^	0.44 ^a^	0.53 ^a^	0.20 ^b^
(*Z*)-2-pentenal	LRI,MS	1093	11.73 ^ab^	7.43 ^c^	16.92 ^a^	8.09 ^bc^	5.29 ^c^	5.70 ^c^
(*E*)-2-pentenal	S,LRI,MS	1115	52.30 ^a^	48.87 ^a^	43.67 ^ab^	39.40 ^ab^	24.74 ^b^	42.38 ^ab^
(*E*)-3-hexenal	LRI,MS	1125	57.10 ^a^	42.81 ^b^	67.22 ^a^	43.82 ^ab^	23.48 ^c^	35.30 ^bc^
(*Z*)-3-hexenal (mg/kg)	LRI,MS	1130	1.36 ^a^	0.40 ^cd^	0.96 ^abc^	1.09 ^ab^	0.47 ^bcd^	0.14 ^d^
heptanal	LRI,MS	1175	4.37	3.60	4.33	3.19	3.03	3.90
(*Z*)-2-hexenal	LRI,MS	1189	44.49	31.63	48.50	33.38	39.16	32.68
(*E*)-2-hexenal (mg/kg)	S,LRI,MS	1205	19.38 ^bc^	22.54 ^b^	21.96 ^b^	11.90 ^cd^	5.92 ^d^	34.95 ^a^
octanal (mg/kg)	S,LRI,MS	1280	0.10 ^a^	0.11 ^a^	0.09 ^a^	0.07 ^b^	0.07 ^b^	0.09 ^a^
(*Z*)-2-heptenal	LRI,MS	1312	10.60	7.65	4.80	11.30	7.66	7.12
(*E*,*E*)-2,4-hexadienal (mg/kg)	LRI,MS	1381	0.27 ^ab^	0.16 ^cd^	0.35 ^a^	0.20 ^bc^	0.07 ^d^	0.13 ^cd^
(*E*,*Z*)-2,4-hexadienal (mg/kg)	LRI,MS	1385	1.56 ^ab^	1.06 ^bc^	2.19 ^a^	0.87 ^cd^	0.38 ^d^	0.62 ^cd^
(*E*)-2-octenal	S,LRI,MS	1417	11.75 ^a^	5.53 ^c^	8.12 ^bc^	10.47 ^ab^	7.17 ^bc^	9.15 ^b^
(*E*,*E*)-2,4-heptadienal	LRI,MS	1448	33.23 ^b^	19.61 ^d^	30.68 ^bcd^	32.10 ^bc^	21.83 ^c^	44.72 ^a^
2-isopropylidene-3-methylhexa-3,5-dienal	MS	1460	0.36 ^bc^	0.33 ^c^	0.65 ^ab^	0.11 ^c^	0.09 ^c^	0.70 ^a^
(*E*,*E*)-2,4-decadienal	LRI,MS	1750	0.65 ^bc^	0.49 ^c^	1.06 ^ab^	1.52 ^a^	0.85 ^b^	0.85 ^b^
(*E*,*Z*)-2,4-decadienal	LRI,MS	1794	0.67 ^b^	0.26 ^c^	0.47 ^bc^	1.13 ^a^	0.93 ^ab^	0.61 ^bc^
tetradecanal	LRI,MS	1909	1.05	0.97	1.04	1.41	0.99	0.62
*ketones*								
3-pentanone (mg/kg)	LRI,MS	962	0.09 ^c^	0.23 ^a^	0.09 ^c^	0.13 ^bc^	0.19 ^ab^	0.08 ^c^
1-penten-3-one (mg/kg)	S,LRI,MS	1008	0.29 ^a^	0.29 ^a^	0.26 ^ab^	0.18 ^bc^	0.12 ^c^	0.22 ^ab^
3-hexen-2-one	MS	1121	0.57 ^b^	0.67 ^a^	0.58 ^b^	0.44 ^c^	0.55 ^b^	0.45 ^c^
2-octanone	LRI,MS	1275	6.12	0.62	0.54	1.55	1.51	0.89
2-methyl-6-methylene-1,7-octadien-3-one	LRI,MS	1303	44.85 ^a^	21.50 ^b^	51.45 ^a^	24.25 ^b^	16.32 ^b^	62.59 ^a^
6-methyl-5-hepten-2-one	LRI,MS	1326	2.15 ^bc^	1.94 ^bc^	2.63 ^abc^	3.02 ^ab^	4.40 ^a^	1.40 ^c^
2-cyclohexene-1,4-dione I	MS	1712	3.62 ^ab^	2.27 ^cd^	5.11 ^a^	3.20 ^bc^	2.05 ^bcd^	1.04 ^d^
2-cyclohexene-1,4-dione II	MS	1803	1.15 ^ab^	0.68 ^cd^	1.57 ^a^	1.06 ^abc^	0.64 ^cd^	0.29 ^d^
(*Z*)-cinerolone	MS	2002	0.20 ^b^	0.13 ^c^	0.19 ^b^	0.04 ^d^	0.03 ^d^	0.28 ^a^
1-(2,6,6-trimethyl-1-cyclohexen-1-yl)-1-penten-3-one	MS	2056	0.18 ^c^	0.25 ^bc^	0.14 ^c^	0.51 ^a^	0.47 ^ab^	0.12 ^c^
4′-ethoxy-2′-hydroxyoctadecanophenone	MS	2081	0.26 ^bc^	0.12 ^c^	0.90 ^a^	0.24 ^bc^	0.26 ^bc^	0.41 ^b^
*alcohols*								
2-methyl-2-propanol	LRI,MS	886	4.98	4.93	8.45	0.03	0.00	4.33
1-methoxy-2-propanol (mg/kg)	MS	917	0.15 ^b^	0.12 ^b^	0.18 ^ab^	0.15 ^b^	0.23 ^a^	0.14 ^b^
3-pentanol	LRI,MS	1097	11.84	16.51	6.53	6.20	15.09	5.92
2-pentanol	LRI,MS	1109	1.04	2.75	0.67	2.76	1.78	1.59
1-penten-3-ol (mg/kg)	LRI,MS	1148	0.18 ^b^	0.28 ^a^	0.19 ^b^	0.14 ^b^	0.19 ^b^	0.18 ^b^
3-methyl-1-butanol	S,LRI,MS	1197	19.96 ^b^	26.70 ^b^	22.74 ^b^	27.21 ^b^	57.71 ^a^	23.80 ^b^
1-pentanol	S,LRI,MS	1239	10.37 ^bc^	16.24 ^ab^	7.86 ^bc^	20.10 ^a^	22.07 ^a^	6.96 ^c^
(*E*)-2-penten-1-ol	S,LRI,MS	1300	62.07	65.28	64.27	43.81	67.92	61.24
(*Z*)-2-penten-1-ol (mg/kg)	S,LRI,MS	1308	0.26 ^a^	0.31 ^a^	0.27 ^a^	0.21 ^b^	0.28 ^a^	0.25 ^ab^
1-hexanol (mg/kg)	S,LRI,MS	1342	0.70	1.21	1.20	1.17	1.58	0.76
(*E*)-3-hexen-1-ol	LRI,MS	1352	27.42 ^ab^	27.45 ^ab^	27.49 ^ab^	37.90 ^a^	46.03 ^a^	14.76 ^b^
(*Z*)-3-hexen-1-ol (mg/kg)	S,LRI,MS	1372	1.63 ^b^	1.08 ^bc^	1.50 ^bc^	2.77 ^a^	1.92 ^ab^	0.61 ^c^
(*E*)-2-hexen-1-ol (mg/kg)	S,LRI,MS	1394	1.00 ^b^	1.68 ^b^	1.37 ^b^	1.05 ^b^	3.42 ^a^	1.42 ^b^
(*Z*)-2-hexen-1-ol	S,LRI,MS	1402	7.53	122.27	11.84	11.20	15.53	10.63
1-heptanol	S,LRI,MS	1445	4.05	2.16	2.23	3.38	3.39	2.71
2-ethyl-1-hexanol	S,LRI,MS	1480	4.58 ^d^	5.90 ^c^	10.39 ^a^	4.32 ^d^	4.01 ^d^	6.93 ^b^
1-octanol	S,LRI,MS	1546	7.65	5.29	5.41	5.56	6.01	5.98
2,4-hexadien-1-ol	LRI,MS	1568	0.11	0.10	0.00	0.65	0.64	0.00
1-nonanol	S,LRI,MS	1650	3.73	2.56	3.14	2.53	3.32	3.10
3-methyl-3-cyclohexen-1-ol	MS	1744	4.66 ^a^	2.34 ^b^	4.39 ^a^	2.25 ^b^	0.65 ^b^	0.88 ^b^
2-(2-butoxyethoxy)-ethanol	LRI,MS	1779	3.83 ^b^	2.43 ^b^	42.91 ^a^	7.86 ^b^	7.62 ^b^	9.91 ^b^
2,2′-oxybis-1-propanol	LRI,MS	1865	4.04	3.46	4.51	2.27	2.59	2.44
tetradecanol	LRI,MS	2158	25.23 ^a^	26.72 ^a^	12.11 ^ab^	31.35 ^a^	13.64 ^ab^	6.70 ^b^
*acids*								
acetic acid (mg/kg)	S,LRI,MS	1427	1.82	2.55	2.46	2.49	4.53	1.70
acid (n.i.) (mg/kg)	MS	1476	0.91 ^ab^	0.82 ^b^	1.17 ^a^	1.10 ^a^	1.18 ^a^	1.07 ^a^
butanoic acid	S,LRI,MS	1602	27.65 ^c^	41.57 ^bc^	40.46 ^bc^	52.13 ^ab^	72.47 ^a^	30.43 ^bc^
hexanoic acid (mg/kg)	S,LRI,MS	1823	0.76 ^c^	1.23 ^bc^	1.32 ^bc^	1.74 ^ab^	2.21 ^a^	1.00 ^c^
2-ethylhexanoic acid	LRI,MS	1925	3.41 ^c^	8.59 ^c^	52.77 ^a^	10.47 ^bc^	12.29 ^bc^	23.46 ^b^
(*E*)-3-hexenoic acid	LRI,MS	1926	12.93 ^b^	9.33 ^bc^	16.87 ^ab^	24.46 ^a^	5.08 ^bc^	1.38 ^c^
octanoic acid (mg/kg)	S,LRI,MS	2033	0.48 ^b^	7.82 ^a^	1.29 ^b^	1.65 ^b^	1.47 ^b^	0.59 ^b^
sorbic acid	LRI,MS	2048	7.83 ^b^	4.94 ^bc^	14.78 ^a^	8.44 ^b^	4.58 ^bc^	1.04 ^c^
nonanoic acid (mg/kg)	S,LRI,MS	2139	41.76 ^b^	434.34 ^a^	54.30 ^b^	41.10 ^b^	55.83 ^b^	29.51 ^b^
decanoic acid (mg/kg)	S,LRI,MS	2244	0.07 ^b^	0.43 ^a^	0.14 ^b^	0.02 ^b^	0.06 ^b^	0.06 ^b^
*esters*								
allyl acetate	LRI,MS	805	53.61	58.55	69.96	43.21	30.69	37.81
methyl acetate	LRI,MS	816	91.00 ^b^	166.56 ^a^	101.38 ^ab^	59.88 ^b^	115.21 ^ab^	81.99 ^b^
1,1,1-trimethoxyethane	MS	871	61.25 ^b^	328.53 ^a^	117.33 ^ab^	50.70 ^b^	85.45 ^b^	24.70 ^b^
ethyl 2-methylbutanoate	LRI,MS	1042	0.90	0.64	0.55	0.70	0.70	0.51
isoamyl acetate	S,LRI,MS	1114	4.91 ^a^	4.92 ^a^	2.26 ^b^	2.88 ^b^	3.19 ^b^	1.69 ^b^
methyl 3-methyl-2-butenoate	LRI,MS	1157	3.79	0.85	2.84	2.61	2.86	1.75
methyl hexanoate	LRI,MS	1178	1.29	1.01	2.05	1.51	1.02	1.29
ethyl hexanoate	S,LRI,MS	1228	0.06	0.23	0.31	0.50	0.35	0.12
hexyl acetate	S,LRI,MS	1265	21.75 ^b^	89.41 ^a^	25.62 ^b^	11.18 ^b^	91.10 ^a^	13.11 ^b^
(*Z*)-3-hexen-1-yl acetate (mg/kg)	S,LRI,MS	1309	0.27 ^b^	0.62 ^a^	0.20 ^b^	0.05 ^b^	0.26 ^b^	0.09 ^b^
(*Z*)-2-hexen-1-yl acetate	LRI,MS	1326	1.52 ^b^	4.02 ^a^	1.44 ^b^	0.91 ^b^	1.75 ^b^	0.91 ^b^
(*E*)-3-hexenyl butanoate	LRI,MS	1454	2.23 ^abc^	0.88 ^c^	2.34 ^ab^	1.47 ^bc^	2.94 ^a^	1.71 ^abc^
3-hydroxy-2,4,4-trimethylpentyl 2-methylpropanoate	MS	1854	0.37 ^c^	0.16 ^c^	4.94 ^a^	1.06 ^bc^	0.95 ^bc^	1.54 ^b^
methyl cinnamoylglycinate	MS	1960	3.44 ^b^	3.34 ^b^	6.22 ^a^	2.62 ^c^	2.25 ^c^	3.33 ^b^
triacetin	LRI,MS	2049	1.09 ^bc^	0.41 ^c^	5.19 ^a^	1.44 ^bc^	1.02 ^bc^	1.77 ^b^
2-propenyl pentanoate	MS	2074	3.07 ^bc^	1.38 ^cd^	7.81 ^a^	3.79 ^b^	2.39 ^bcd^	0.80 ^d^
methyl 3-oxo-2-pentyl-cyclopentaneacetate	MS	2257	7.59 ^ab^	3.69 ^c^	12.29 ^a^	8.44 ^ab^	8.06 ^ab^	6.03 ^bc^
*benzenoids*								
benzene	LRI,MS	926	9.83	14.67	14.44	12.05	11.62	10.10
toluene (mg/kg)	LRI,MS	1027	0.10	0.17	0.07	0.40	0.17	0.10
*m*-xylene	LRI,MS	1122	22.55	19.69	15.30	105.52	26.14	17.65
*p*-xylene (mg/kg)	LRI,MS	1128	0.06	0.06	0.05	0.22	0.07	0.05
*o*-xylene (mg/kg)	LRI,MS	1172	0.03	0.03	0.02	0.11	0.04	0.03
*p*-ethyltoluene	LRI,MS	1213	16.73	17.37	11.75	56.70	14.44	11.29
1,3,5-trimethylbenzene (mesitylene)	LRI,MS	1235	11.77	11.52	12.45	32.07	9.69	8.84
2-ethyltoluene	LRI,MS	1251	7.00	6.28	5.39	18.11	5.10	4.07
*p*-cymene	S,LRI,MS	1262	1.51	1.57	2.15	2.87	31.68	4.46
*m*-cymene	LRI,MS	1270	19.46	16.99	16.47	40.75	10.97	10.45
1,3-diethylbenzene	LRI,MS	1293	1.54	0.75	0.84	2.14	0.59	0.71
*o*-cymene	LRI,MS	1296	1.64	1.38	1.37	3.62	0.94	1.09
1-ethyl-3,5-dimethylbenzene	LRI,MS	1316	4.25	4.17	3.80	8.75	2.79	3.11
1,2,3-trimethylbenzene (hemellitol)	LRI,MS	1324	8.25	6.56	7.35	14.88	4.70	4.04
anisole	LRI,MS	1329	3.77	2.45	2.91	1.27	3.42	1.85
1,2,4,5-tetramethylbenzene	LRI,MS	1413	1.89	1.36	1.44	3.99	1.13	1.09
isodurene	LRI,MS	1422	2.78	2.21	2.22	5.44	2.06	1.80
1,2,3,4-tetramethylbenzene	MS	1423	2.78	2.20	2.20	5.39	2.01	1.81
*p*-cymenene	LRI,MS	1436	9.05 ^b^	7.21 ^c^	13.45 ^a^	7.49 ^c^	6.48 ^c^	6.87 ^c^
methyl benzoate	LRI,MS	1601	14.74 ^b^	3.88 ^c^	22.08 ^b^	41.94 ^a^	13.97 ^b^	4.01 ^c^
acetophenone	S,LRI,MS	1626	14.76 ^b^	13.46 ^b^	20.73 ^a^	13.08 ^b^	12.68 ^b^	13.60 ^b^
estragole	LRI,MS	1655	0.06 ^c^	0.03 ^c^	0.02 ^c^	0.90 ^b^	1.40 ^a^	0.01 ^c^
4-ethylbenzaldehyde	LRI,MS	1689	6.13 ^b^	4.73 ^c^	9.68 ^a^	4.66 ^c^	3.77 ^c^	4.66 ^c^
methyl salicylate	LRI,MS	1755	47.45 ^ab^	18.17 ^d^	62.13 ^a^	61.68 ^a^	37.24 ^bc^	30.00 ^c^
aromatic aldehyde I (n.i.)	MS	1810	9.56 ^b^	9.57 ^b^	18.54 ^a^	6.75 ^c^	5.56 ^c^	9.18 ^c^
aromatic aldehyde II (n.i.)	MS	1839	9.12 ^b^	9.29 ^b^	18.39 ^a^	6.46 ^c^	5.50 ^c^	8.81 ^c^
benzyl alcohol	S,LRI,MS	1851	20.21 ^c^	22.03 ^c^	24.09 ^bc^	23.65 ^bc^	35.74 ^ab^	39.17 ^a^
2-phenylethyl alcohol (mg/kg)	S,LRI,MS	1885	0.33	0.30	0.39	0.35	0.32	0.38
benzyl nitrile	LRI,MS	1896	0.04 ^b^	0.00 ^b^	0.16 ^b^	0.00 ^b^	0.00 ^b^	1.17 ^a^
lilial	LRI,MS	2020	1.55 ^bc^	1.58 ^bc^	1.04 ^bc^	2.42 ^ab^	3.74 ^a^	1.01 ^c^
methyl 2-methoxybenzoate	LRI,MS	2037	5.21 ^a^	0.43 ^c^	4.21 ^ab^	3.49 ^a b^	2.45 ^bc^	1.19 ^c^
complex benzenoid (n.i.)	MS	2041	3.94 ^b^	5.40 ^b^	3.69 ^b^	12.24 ^a^	11.25 ^a^	3.07 ^b^
2-phenoxyethanol	LRI,MS	2106	3.30 ^bc^	2.07 ^c^	4.99 ^bc^	10.29 ^a^	7.11 ^ab^	5.49 ^bc^
methyl anthranilate	LRI,MS	2200	0.73 ^b^	0.05 ^c^	0.76 ^b^	1.15 ^b^	2.04 ^a^	0.01 ^c^
menthyl salicylate	MS	2273	1.25 ^b^	2.57 ^a^	2.16 ^ab^	2.74 ^a^	2.27 ^a^	1.39 ^b^
4-ethoxystyrene	MS	2348	1.97 ^c^	1.96 ^c^	2.20 ^bc^	3.07 ^a^	2.74 ^ab^	1.95 ^c^
benzoic acid	LRI,MS	2381	11.32 ^b^	44.78 ^a^	38.56 ^a^	5.62 ^b^	7.75 ^b^	14.51 ^b^
*furanoids*								
2-ethylfuran (mg/kg)	LRI,MS	938	0.14 ^b^	0.08 ^cd^	0.22 ^a^	0.09 ^bc^	0.05 ^cd^	0.03 ^d^
2-vinylfuran	LRI,MS	1059	10.26 ^b^	5.89 ^bc^	17.85 ^a^	9.15 ^b^	5.32 ^bc^	1.94 ^c^
4-methyl-2,3-dihydrofuran	LRI,MS	1184	4.98 ^ab^	4.59 ^b^	3.83 ^b^	6.13 ^a^	4.90 ^ab^	3.86 ^b^
2-pentylfuran	LRI,MS	1225	7.81	7.60	7.33	8.82	10.79	62.68
5-methyl-2-furancarboxaldehyde	LRI,MS	1551	4.99 ^b^	3.33 ^c^	7.19 ^a^	3.58 ^bc^	1.90 ^cd^	1.55 ^d^
2(5H)-furanone	LRI,MS	1722	5.90	3.67	5.23	2.23	1.42	2.94
5-ethyl-2(5H)-furanone (mg/kg)	LRI,MS	1733	0.19 ^a^	0.08 ^bc^	0.19 ^a^	0.15 ^ab^	0.06 ^bc^	0.03 ^c^
2-ethyl-5-methyl-tetrahydrofuran (mg/kg)	MS	1933	0.44 ^ab^	0.21 ^cd^	0.58 ^a^	0.34 ^bc^	0.15 ^cd^	0.07 ^d^
*miscellaneous*								
dimethyl sulfide	LRI,MS	739	1.21 ^ab^	1.26 ^ab^	2.49 ^a^	1.18 ^ab^	0.74 ^b^	1.81 ^ab^
2,4-dihydro-5-methyl-3H-pyrazol-3-one I	MS	978	0.90	2.14	1.10	10.00	4.62	0.38
1,2-dihydro-5-methyl-3H-pyrazol-3-one	MS	1109	0.85	1.17	0.52	4.51	2.12	0.09
2,4-dihydro-5-methyl-3H-pyrazol-3-one II	MS	1150	0.08	0.35	0.09	1.82	0.60	0.01
2-phenyl-1H-indole	MS	1502	78.56 ^c^	93.8 ^b^	139.59 ^a^	67.51 ^c^	74.11 ^c^	95.38 ^b^
dimethyl sulfoxide	LRI,MS	1540	26.39 ^abc^	15.52 ^c^	27.62 ^ab^	32.02 ^a^	23.96 ^abc^	16.65 ^bc^
n.i. (*m*/*z* 189,207,131)	MS	1688	0.51 ^a^	0.21 ^b^	0.45 ^ab^	0.47 ^a^	0.25 ^ab^	0.19 ^b^
n.i. (*m*/*z* 84,85,41,42,39,133,147,175)	MS	1990	1.91 ^b^	1.20 ^b^	3.72 ^a^	1.07 ^b^	1.32 ^b^	1.21 ^b^
phenol	S,LRI,MS	1972	5.27 ^c^	6.01 ^b^	9.88 ^a^	4.38 ^d^	4.16 ^d^	5.95 ^b^
*totals (mg/kg)*								
total hydrocarbons			2.12 ^a^	1.54 ^b^	1.99 ^a^	1.13 ^c^	1.01 ^c^	1.70 ^b^
total monoterpenes			0.35 ^b^	0.23 ^b^	0.41 ^b^	0.71 ^b^	1.85 ^a^	0.26 ^b^
total sesquiterpenes			12.36 ^a^	10.15 ^a^	9.29 ^a^	12.54 ^a^	3.54 ^b^	2.66 ^b^
total aldehydes			23.19 ^b^	24.78 ^b^	26.10 ^b^	14.79 ^c^	7.64 ^c^	36.37 ^a^
total ketones			0.44 ^b^	0.56 ^a^	0.41 ^bc^	0.34 ^c^	0.33 ^c^	0.37 ^c^
total alcohols			4.12 ^bc^	5.02 ^bc^	4.95 ^bc^	5.69 ^ab^	7.89 ^a^	3.52 ^c^
total acids			45.85 ^b^	447.25 ^a^	60.81 ^b^	48.19 ^b^	65.37 ^b^	33.99 ^b^
total esters			0.52 ^b^	1.29 ^a^	0.56 ^b^	0.25 ^b^	0.61 ^b^	0.27 ^b^
total benzenoids			0.80	0.81	0.87	1.60	0.88	0.79
total furanoids			0.80	0.40	1.04	0.61	0.28	0.76
total miscellaneous			0.12 ^b^	0.12 ^b^	0.19 ^a^	0.12 ^b^	0.11 ^b^	0.12 ^b^

ID—identification of compounds; S—retention time and mass spectrum consistent with that of the pure standard and with NIST05 mass spectra electronic library; LRI—linear retention index consistent with that found in literature; MS—mass spectra consistent with that from NIST05 mass spectra electronic library or literature; n.i.—not identified. The compounds with only MS symbol in ID column were tentatively identified. The compounds for which pure standards were not available (without symbol S in the ID column) were quantified semi-quantitatively and their concentrations were expressed as equivalents of compounds with similar chemical structure assuming a response factor = 1. Different superscript lowercase letters in a row represent statistically significant differences between mean values at *p* < 0.05 obtained by one-way ANOVA and least significant difference (LSD) test.

**Table 2:** Sensory descriptors and odor perception thresholds of volatile aroma compounds in monovarietal extra virgin olive oils produced from Buža, Istarska bjelica, Rosinjola, Oblica, Lastovka, and Leccino varieties in Croatia, sorted in descending order according to their average odor activity values (OAV).

Volatile Compound	Sensory Descriptor (Aroma) *	Threshold *	Odor Activity Value (OAV)					
			Buža	I. bjelica	Rosinjola	Oblica	Lastovka	Leccino
*OAV* > *1*								
(*Z*)-3-hexenal	leaf-like, green, apple-like	1.7	**800.00 ^a^**	235.29 ^cd^	564.71 ^abc^	641.18 ^ab^	276.47 ^bcd^	82.35 ^d^
1-penten-3-one	leaf, green, pungent, sweet	0.73	**397.26 ^a^**	**397.26 ^a^**	356.16 ^ab^	246.58 ^bc^	164.38 ^c^	301.37 ^ab^
(*E*)-2-hexenal	green, apple-like, bitter almond	420	46.14 ^bc^	53.67 ^b^	52.29 ^b^	28.33 ^cd^	14.10 ^d^	**83.21 ^a^**
hexanal	green, sweet, green apple, grassy	75	3.20 ^b^	3.87 ^b^	3.60 ^b^	5.87 ^a^	**7.07 ^a^**	2.67 ^b^
3-methylbutanal	malty	5.2	2.02 ^b^	**4.29 ^a^**	2.05 ^b^	1.63 ^b^	2.37 ^b^	2.36 ^b^
1-hexanol	fruit, banana, soft, grass	400	1.75	3.03	3.00	2.93	**3.95**	1.90
2-methylbutanal	malty	5.4	1.99	2.48	2.21	1.46	**3.46**	2.55
hexanoic acid	pungent, rancid, sweaty	700	1.09 ^c^	1.76 ^bc^	1.89 ^bc^	2.49 ^ab^	**3.16 ^a^**	1.43 ^c^
(*Z*)-3-hexen-1-yl acetate	green, banana-like, olive fruity	200	1.35 ^b^	**3.10 ^a^**	1.00 ^b^	0.25 ^b^	1.30 ^b^	0.45 ^b^
(*E*)-2-octenal	herbaceous, spicy	4	**2.94 ^a^**	1.38 ^c^	2.03 ^bc^	2.62 ^ab^	1.79 ^bc^	2.29 ^b^
octanoic acid	oily, fatty	3000	0.16 ^b^	**2.61 ^a^**	0.43 ^b^	0.55 ^b^	0.49 ^b^	0.20 ^b^
(*Z*)-3-hexen-1-ol	green, apple, leaf-like, banana	1100	1.48 ^b^	0.98 ^bc^	1.36 ^bc^	**2.52 ^a^**	1.75 ^ab^	0.55 ^c^
ethyl 2-methylbutanoate	fruity	0.72	**1.25**	0.89	0.76	0.97	0.97	0.71
*OAV* < *1*								
1-penten-3-ol	lawn, olive, leaf, pungent	400	0.45 ^b^	**0.70 ^a^**	0.48 ^b^	0.35 ^b^	0.48 ^b^	0.45 ^b^
(*E*)-2-hexen-1-ol	green, grass, leaves, sweet	5000	0.20 ^b^	0.34 ^b^	0.27 ^b^	0.21 ^b^	**0.68 ^a^**	0.28 ^b^
3-methyl-1-butanol	woody, whiskey, sweet	100	0.20 ^b^	0.27 ^b^	0.23 ^b^	0.27 ^b^	**0.58 ^a^**	0.24 ^b^
octanal	fatty, sharp, citrus-like, soapy	320	0.31 ^a^	**0.34 ^a^**	0.28 ^a^	0.22 ^b^	0.22 ^b^	0.28 ^a^
(*E*)-2-penten-1-ol	green fruity, fresh olive fruits	250	0.25	0.26	0.26	0.18	**0.27**	0.24
(*E*)-2-pentenal	green, apple, bitter almond	300	**0.17 ^a^**	0.16 ^a^	0.15 ^ab^	0.13 ^ab^	0.08 ^b^	0.14 ^ab^
butanoic acid	rancid, cheese	650	0.04 ^c^	0.06 ^bc^	0.06 ^bc^	0.08 ^ab^	**0.11 ^a^**	0.05 ^bc^
(*E*,*Z*)-2,4-decadienal	deep-fried	10	0.07 ^b^	0.03 ^c^	0.05 ^bc^	**0.11 ^a^**	0.09 ^ab^	0.06 ^bc^
hexyl acetate	green, fruity, sweet, apple	1040	0.02 ^b^	**0.09 ^a^**	0.02 ^b^	0.01 ^b^	**0.09 ^a^**	0.01 ^b^
1-pentanol	fruity, strong, sticky, balsamic	470	0.02 ^bc^	0.03 ^ab^	0.02 ^bc^	0.04 ^a^	**0.05 ^a^**	0.01 ^c^
octane	sweety, alcane	940	0.03	**0.04**	0.03	0.03	**0.04**	0.03
2-octanone	mould, green	510	**0.01**	0.00	0.00	0.00	0.00	0.00
(*E*)-3-hexen-1-ol	green, bitter	1500	0.02 ^ab^	0.02 ^ab^	0.02 ^ab^	**0.03 ^a^**	**0.03 ^a^**	0.01 ^b^
1-nonanol	fatty, rancid	280	0.01	0.01	0.01	0.01	0.01	0.01
heptanal	oily, fatty, woody	500	0.01	0.01	0.01	0.01	0.01	0.01
(*E*,*E*)-2,4-heptadienal	fatty, rancid	3620	0.01 ^b^	0.01 ^d^	0.01 ^bcd^	0.01 ^bc^	0.01 ^c^	0.01 ^a^
(*E*,*E*)-2,4-decadienal	deep-fried	180	0.00 ^bc^	0.00 ^c^	0.01 ^ab^	0.01 ^a^	0.00 ^b^	0.00 ^b^
6-methyl-5-hepten-2-one	pungent, green	1000	0.00 ^bc^	0.00 ^bc^	0.00 ^abc^	0.00 ^ab^	0.00 ^a^	0.00 ^c^
3-pentanone	fruity, green, sweet	70,000	0.00 ^c^	0.00 ^a^	0.00 ^c^	0.00 ^bc^	0.00 ^ab^	0.00 ^c^

* sensory descriptors and odor perception thresholds (µg/kg oil) reported in literature [21,44,45,46,47]. Values in bold indicate the highest average OAV for a given volatile compound among monovarietal extra virgin olive oils.

**Table 3:** Concentrations (mg/kg) of phenols determined by ultra-performance liquid chromatography with diode-array detection (UPLC-DAD) in monovarietal extra virgin olive oils produced from Buža, Istarska bjelica, Rosinjola, Oblica, Lastovka, and Leccino varieties in Croatia.

Phenol	Variety					
	Buža	I. bjelica	Rosinjola	Oblica	Lastovka	Leccino
*simple phenols*						
tyrosol	4.87 ^b^	11.29 ^a^	3.69 ^b^	9.10 ^ab^	12.28 ^a^	5.60 ^b^
hydroxytyrosol	5.40 ^c^	10.21 ^b^	5.59 ^bc^	6.33 ^bc^	20.17 ^a^	6.47 ^bc^
hydroxytyrosol acetate *	0.35 ^c^	0.67 ^b^	0.37 ^bc^	0.42 ^bc^	1.44 ^a^	0.50 ^bc^
vanillin	0.21 ^b^	0.16 ^bc^	0.20 ^bc^	0.12 ^c^	0.11 ^c^	0.31 ^a^
*phenolic acids*						
vanillic acid	0.31	0.32	0.33	0.18	0.37	0.25
*p*-coumaric acid	1.26 ^bc^	0.90 ^c^	0.82 ^cd^	1.69 ^b^	2.80 ^a^	0.34 ^d^
flavonoids						
luteolin	2.02 ^bc^	2.95 ^a^	2.86 ^ab^	2.93 ^a^	3.35 ^a^	1.89 ^c^
apigenin	0.55 ^bc^	0.87 ^a^	0.66 ^b^	0.33 ^d^	0.39 ^d^	0.46 ^cd^
*lignans*						
pinoresinol	9.97 ^a^	4.02 ^c^	6.98 ^b^	3.21 ^c^	3.68 ^c^	4.14 ^c^
Acetoxypinoresinol *	6.72 ^c^	14.11 ^a^	11.39 ^ab^	8.69 ^bc^	11.94 ^ab^	7.49 ^c^
*secoiridoids*						
Secologanoside *	0.03 ^b^	0.04 ^b^	0.03 ^b^	0.04 ^b^	0.06 ^a^	0.04 ^b^
elenolic acid glucoside *	0.04 ^bc^	0.04 ^c^	0.05 ^abc^	0.05 ^ab^	0.06 ^a^	0.04 ^c^
3,4-DHPEA-EDA *	95.50 ^b^	115.68 ^b^	104.93 ^b^	98.46 ^b^	121.33 ^b^	175.06 ^a^
oleuropein aglycone I *	72.56 ^bc^	94.57 ^ab^	109.85 ^a^	49.09 ^cd^	115.05 ^a^	41.17 ^d^
*p*-HPEA-EDA *	49.15 ^b^	82.70 ^a^	47.35 ^b^	76.79 ^a^	49.21 ^b^	87.49 ^a^
oleuropein + ligstroside aglycones I & II *	43.38 ^b^	97.82 ^a^	49.19 ^b^	38.63 ^b^	49.33 ^b^	30.11 ^b^
oleuropein aglycone II *	64.44 ^c^	79.72 ^abc^	100.61 ^a^	42.38 ^d^	94.14 ^ab^	71.77 ^bc^
ligstroside aglycon III *	1.66 ^c^	4.60 ^a^	1.82 ^c^	2.79 ^bc^	1.99 ^c^	4.04 ^ab^
oleuropein aglycone III *	9.06 ^c^	15.84 ^a^	11.66 ^bc^	11.40 ^bc^	13.75 ^ab^	9.85 ^c^
total phenols	367.25 ^c^	536.49 ^a^	458.38 ^abc^	352.63 ^c^	501.45 ^ab^	447.00 ^bc^

* The phenols for which pure standards were not available were quantified semi-quantitatively and their concentrations were expressed as equivalents of phenols with similar chemical structure assuming a response factor = 1. Different superscript lowercase letters in a row represent statistically significant differences between mean values at *p* < 0.05 obtained by one-way ANOVA and least significant difference (LSD) test.

## Supplementary Materials

The following are available online at https://www.mdpi.com/2304-8158/8/11/565/s1, Table S1: Climate parameters in the Istria and Dalmatia regions of Croatia in 2015, Table S2: Standardized coefficients of the variables selected for the differentiation of monovarietal Buža, Istarska bjelica, Rosinjola, Oblica, Lastovka, and Leccino extra virgin olive oils on the first three discriminant functions obtained by stepwise linear discriminant analysis, and the percentage of correct classification at each step, Table S3: The intensities and scores of the sensory attributes perceived in monovarietal extra virgin olive oils produced from Buža, Istarska bjelica, Rosinjola, Oblica, Lastovka, and Leccino varieties in Croatia.
