# Supplementary material for: Inter-Varietal Diversity of Typical Volatile and Phenolic Profiles of Croatian Extra Virgin Olive Oils as Revealed by GC-IT-MS and UPLC-DAD Analysis
Source: Foods. 2019 Nov 9;8(11):565. doi: 10.3390/foods8110565 (PMC6915403; doi:10.3390/foods8110565)
Supplement: Supplementary file 1 [file foods-08-00565-s001.zip › Table S1 - Lukic et al 2019.docx]

Table S1. Climate parameters in the Istria and Dalmatia regions of Croatia in 2015

| Parameter | Region | Month (2015) | | | | | | | | | | | |
| --- | --- | --- | --- | --- | --- | --- | --- | --- | --- | --- | --- | --- | --- |
|  |  | I | II | III | IV | V | VI | VII | VII | IX | X | XI | XII |
| Temp. (°C) | Istria | 6.4 | 5.7 | 9.0 | 12.0 | 17.6 | 22.1 | 26.3 | 23.9 | 19.2 | 13.7 | 9.3 | 6.8 |
|  | Dalmatia | 9.1 | 8.9 | 11.4 | 14.2 | 19.9 | 23.9 | 28.3 | 26.8 | 22.4 | 17.3 | 13.4 | 10.9 |
| Rainfall (mm) | Istria | 51.3 | 53.5 | 44.8 | 23.8 | 37.7 | 51.7 | 22.7 | 77.5 | 64.0 | 162.8 | 28.0 | 0.5 |
|  | Dalmatia | 93.8 | 157.5 | 61.3 | 47.8 | 66.1 | 37.4 | 9.2 | 73.8 | 68.8 | 241.4 | 45.1 | 0.1 |
