# Supplementary material for: Inter-Varietal Diversity of Typical Volatile and Phenolic Profiles of Croatian Extra Virgin Olive Oils as Revealed by GC-IT-MS and UPLC-DAD Analysis
Source: Foods. 2019 Nov 9;8(11):565. doi: 10.3390/foods8110565 (PMC6915403; doi:10.3390/foods8110565)
Supplement: Supplementary file 1 [file foods-08-00565-s001.zip › Table S2 - Lukic et al 2019.docx]

Table S2. The intensities and scores of the sensory attributes perceived in monovarietal extra virgin olive oils produced from Buža, Istarska bjelica, Rosinjola, Oblica, Lastovka, and Leccino varieties in Croatia

| sensory attribute | variety | | | | | |
| --- | --- | --- | --- | --- | --- | --- |
|  | Buža | I. bjelica | Rosinjola | Oblica | Lastovka | Leccino |
| fruity | 5.51^a^ | 5.53^a^ | 5.84^a^ | 5.00^ab^ | 4.70^b^ | 5.34^a^ |
| green grass/leaves | 4.24^a^ | 4.40^a^ | 4.36^a^ | 3.12^bc^ | 2.64^c^ | 3.42^b^ |
| apple | 1.67^ab^ | 1.05^c^ | 1.36^abc^ | 1.97^a^ | 0.00^d^ | 1.32^bc^ |
| tomato | 1.28^a^ | 0.78^ab^ | 1.43^a^ | 1.11^a^ | 0.26^b^ | 1.06^a^ |
| almond | 2.74^a^ | 1.15^c^ | 1.63^bc^ | 1.94^b^ | 2.65^a^ | 2.08^b^ |
| aromatic herbs | 4.01^a^ | 2.86^a^ | 3.16^a^ | 2.43^bc^ | 2.01^c^ | 2.69^ab^ |
| chicory/rocket | 3.98^a^ | 2.79^b^ | 3.03^b^ | 0.78^c^ | 0.00^c^ | 0.72^c^ |
| green banana | 0.39^bc^ | 0.00^c^ | 0.14^bc^ | 1.45^a^ | 0.73^ab^ | 0.82^ab^ |
| wood | 0.00^b^ | 0.00^b^ | 0.00^b^ | 0.00^b^ | 1.36^a^ | 0.00^b^ |
| bitter | 5.00^bcd^ | 5.54^a^ | 5.19^abc^ | 4.56^d^ | 5.43^ab^ | 4.84^cd^ |
| pungent | 5.74^b^ | 6.54^a^ | 6.33^a^ | 5.16^c^ | 4.00^d^ | 4.84^c^ |
| sweet | 1.25^b^ | 0.48^c^ | 0.73^bc^ | 1.84^a^ | 0.68^c^ | 1.90^a^ |
| astringent | 1.83^a^ | 2.19^a^ | 1.11^b^ | 1.03^b^ | 2.17^a^ | 0.68^b^ |
| complexity | 7.54^a^ | 6.73^b^ | 7.47^a^ | 7.55^a^ | 7.45^a^ | 7.75^a^ |
| harmony | 7.84^a^ | 6.93^c^ | 7.81^a^ | 7.59^ab^ | 7.03^bc^ | 7.85^a^ |
| persistency | 8.16^b^ | 8.72^a^ | 8.34^ab^ | 7.52^c^ | 7.75^bc^ | 6.90^d^ |
| overall quality | 8.01 | 7.91 | 7.98 | 7.85 | 7.60 | 7.88^ns^ |
