# Supplementary material for: Inter-Varietal Diversity of Typical Volatile and Phenolic Profiles of Croatian Extra Virgin Olive Oils as Revealed by GC-IT-MS and UPLC-DAD Analysis
Source: Foods. 2019 Nov 9;8(11):565. doi: 10.3390/foods8110565 (PMC6915403; doi:10.3390/foods8110565)
Supplement: Supplementary file 1 [file foods-08-00565-s001.zip › Table S3 - Lukic et al 2019.docx]

Table S3. Standardized coefficients of the variables selected for the differentiation of monovarietal Buža, Istarska bjelica, Rosinjola, Oblica, Lastovka, and Leccino extra virgin olive oils on the first three discriminant functions obtained by stepwise linear discriminant analysis, and the percentage of correct classification at each step.

| no. | variable | discriminant function | | |  | correct classification (%) | | | | | | |
| --- | --- | --- | --- | --- | --- | --- | --- | --- | --- | --- | --- | --- |
|  |  | root 1 | root 2 | root 3 |  | Buža | I. bjelica | Rosinjola | Oblica | Lastovka | Leccino | Total |
| **1** | **phenol** | **0.14** | **1.24** | **-0.24** |  | **31.58** | **68.18** | **75.00** | **73.33** | **0.00** | **0.00** | **41.76** |
| **2** | **nonanoic acid** | **-0.16** | **0.83** | **-0.21** |  | **42.11** | **72.73** | **87.50** | **66.67** | **0.00** | **58.82** | **56.04** |
| **3** | **α-muurolene** | **1.80** | **-0.97** | **0.80** |  | **68.42** | **77.27** | **75.00** | **93.33** | **70.00** | **88.24** | **79.12** |
| **4** | **3,7-decadiene I** | **-0.36** | **-0.48** | **-0.09** |  | **78.95** | **90.91** | **100.00** | **100.00** | **80.00** | **88.24** | **89.01** |
| **5** | **estragole** | **0.72** | **0.16** | **-0.41** |  | **89.47** | **90.91** | **100.00** | **100.00** | **100.00** | **94.12** | **94.51** |
| 6 | sesquiterpene II (n.i.) | -1.41 | 0.74 | 0.02 |  | 94.74 | 90.91 | 100.00 | 93.33 | 100.00 | 94.12 | 94.51 |
| 7 | (*Z*)-2-pentene | -0.31 | 0.33 | -0.26 |  | 94.74 | 90.91 | 100.00 | 93.33 | 100.00 | 88.24 | 93.41 |
| 8 | 2-ethyl-5-methyl-tetrahydrofuran | 0.00 | -0.28 | 1.97 |  | 94.74 | 90.91 | 100.00 | 93.33 | 100.00 | 88.24 | 93.41 |
| 9 | methyl salicylate | 0.63 | -0.65 | -0.01 |  | 94.74 | 90.91 | 100.00 | 93.33 | 100.00 | 94.12 | 94.51 |
| 10 | hexyl acetate | 0.21 | 0.45 | -0.17 |  | 94.74 | 95.45 | 100.00 | 93.33 | 100.00 | 94.12 | 95.60 |
| 11 | sesquiterpene IV (n.i.) | 0.71 | -0.09 | -0.30 |  | 94.74 | 95.45 | 100.00 | 93.33 | 100.00 | 94.12 | 95.60 |
| 12 | methyl 2-methoxybenzoate | 0.09 | -0.16 | 0.14 |  | 89.47 | 95.45 | 100.00 | 93.33 | 100.00 | 94.12 | 94.51 |
| 13 | γ-elemene | 0.33 | 0.26 | -0.62 |  | 89.47 | 95.45 | 100.00 | 100.00 | 100.00 | 100.00 | 96.70 |
| 14 | methyl cinnamoylglycinate | -1.60 | -1.97 | -1.26 |  | 89.47 | 95.45 | 100.00 | 100.00 | 100.00 | 100.00 | 96.70 |
| 15 | monoterpene I (n.i.) | 0.17 | 0.23 | 0.24 |  | 89.47 | 95.45 | 100.00 | 100.00 | 100.00 | 100.00 | 96.70 |
| 16 | 3-propylcyclohexene | -0.51 | -0.36 | -0.21 |  | 94.74 | 100.00 | 100.00 | 100.00 | 100.00 | 100.00 | 98.90 |
| 17 | (+)-cycloisosativene | -0.26 | 0.14 | -0.06 |  | 89.47 | 100.00 | 100.00 | 100.00 | 100.00 | 100.00 | 97.80 |
| 18 | hexanoic acid | 0.43 | 0.21 | -0.29 |  | 94.74 | 100.00 | 100.00 | 100.00 | 100.00 | 100.00 | 98.90 |
| 19 | γ-terpinene | 0.37 | 0.33 | -0.20 |  | 94.74 | 100.00 | 100.00 | 100.00 | 100.00 | 100.00 | 98.90 |
| 20 | dodecene | 0.30 | 0.12 | -0.17 |  | 94.74 | 100.00 | 100.00 | 100.00 | 100.00 | 100.00 | 98.90 |
| 21 | (*E*,*Z*)-2,4-hexadienal | -0.55 | 0.86 | 2.21 |  | 94.74 | 100.00 | 100.00 | 100.00 | 100.00 | 100.00 | 98.90 |
| 22 | 3-pentanone | -0.25 | 0.62 | 0.41 |  | 100.00 | 100.00 | 100.00 | 100.00 | 100.00 | 100.00 | 100.00 |
| 23 | sorbic acid | 0.60 | 0.28 | -1.98 |  | 100.00 | 100.00 | 100.00 | 100.00 | 100.00 | 100.00 | 100.00 |
| 24 | 3-methyl-1-butanol | -0.03 | -0.67 | -0.21 |  | 100.00 | 100.00 | 100.00 | 100.00 | 100.00 | 100.00 | 100.00 |
| 25 | n.i. (m/z 84,85,41,42,39,133,147,175) | -0.26 | -0.37 | -0.18 |  | 100.00 | 100.00 | 100.00 | 100.00 | 100.00 | 100.00 | 100.00 |
| 26 | acrolein | -0.03 | -0.40 | 0.28 |  | 100.00 | 100.00 | 100.00 | 100.00 | 100.00 | 100.00 | 100.00 |
| 27 | (*Z*)-3-hexenal | 1.18 | 0.84 | -1.42 |  | 100.00 | 100.00 | 100.00 | 100.00 | 100.00 | 100.00 | 100.00 |
| 28 | α-farnesene | -0.54 | -0.12 | 0.19 |  | 100.00 | 100.00 | 100.00 | 100.00 | 100.00 | 100.00 | 100.00 |
| 29 | isoamyl acetate | -0.26 | -0.11 | 0.34 |  | 100.00 | 100.00 | 100.00 | 100.00 | 100.00 | 100.00 | 100.00 |
| 30 | (*E*,*E*)-2,4-decadienal | 0.25 | -0.09 | 0.06 |  | 100.00 | 100.00 | 100.00 | 100.00 | 100.00 | 100.00 | 100.00 |
